# Srlp is crucial for the self-renewal and differentiation of germline stem cells via RpL6 signals in *Drosophila* testes

**DOI:** 10.1038/s41419-019-1527-z

**Published:** 2019-04-01

**Authors:** Jun Yu, Yidan Yan, Xiaojin Luan, Chen Qiao, Yuanyuan Liu, Dan Zhao, Bing Xie, Qianwen Zheng, Min Wang, Wanyin Chen, Cong Shen, Zeyu He, Xing Hu, Xiaoyan Huang, Hong Li, Qixiang Shao, Xia Chen, Bo Zheng, Jie Fang

**Affiliations:** 1 Department of Gynecology, The Affiliated Hospital of Jiangsu University, Jiangsu University, Zhenjiang Jiangsu, 212001 China; 2 Reproductive Sciences Institute of Jiangsu University, Zhenjiang Jiangsu, 212001 China; 3 Department of Clinical Pharmacy, the Affiliated Hospital of Jiangsu University, Jiangsu University, Zhenjiang Jiangsu, 212001 China; 4 Center for Reproduction and Genetics, Suzhou Municipal Hospital, The Affiliated Suzhou Hospital of Nanjing Medical University, Suzhou Jiangsu, 215002 China; 5 Center for Reproduction, The Fourth People’s Hospital of Zhenjiang, Zhenjiang Jiangsu, 212013 China; 6 Department of Clinical Medicine, China Medical University, Shenyang Liaoning, 110001 China; 7 State Key Laboratory of Reproductive Medicine, Department of Histology and Embryology, Nanjing Medical University, Nanjing Jiangsu, 211166 China; 8 Department of Immunology and Jiangsu Key Laboratory of Medical Science and Laboratory Medicine, School of Medicine, Jiangsu University, Zhenjiang Jiangsu, 212013 China

## Abstract

Self-renewal and differentiation in germline stem cells (GSCs) are tightly regulated by the stem cell niche and via multiple approaches. In our previous study, we screened the novel GSC regulatory gene *Srlp* in *Drosophila* testes. However, the underlying mechanistic links between Srlp and the stem cell niche remain largely undetermined. Here, using genetic manipulation of the *Drosophila* model, we systematically analyze the function and mechanism of Srlp in vivo and in vitro. In *Drosophila*, *Srlp* is an essential gene that regulates the self-renewal and differentiation of GSCs in the testis. In the in vitro assay, Srlp is found to control the proliferation ability and cell death in S2 cells, which is consistent with the phenotype observed in *Drosophila* testis. Furthermore, results of the liquid chromatography-tandem mass spectrometry (LC-MS/MS) reveal that RpL6 binds to Srlp. Srlp also regulates the expression of spliceosome and ribosome subunits and controls spliceosome and ribosome function via RpL6 signals. Collectively, our findings uncover the genetic causes and molecular mechanisms underlying the stem cell niche. This study provides new insights for elucidating the pathogenic mechanism of male sterility and the formation of testicular germ cell tumor.

## Introduction

Stem cells are undifferentiated populations with the remarkable potential of self-renewal and differentiation. The stem cell niche, a key microenvironment that regulates stem cell behaviors, supports two distinct adult stem cell populations: germline stem cells (GSCs) and cyst stem cells (CySCs)^1–3^. In *Drosophila* testes, GSCs asymmetrically divide to generate one cell that retains stemness and a gonialblast that proliferates and differentiates^2^. The gonialblast undergoes four rounds of transit-amplifying (TA) spermatogonial divisions to generate a 16-cell spermatogonia cluster in which individual germ cells are connected by ring canals and a branched fusome^4^. Somatic cells, including apical hubs and CySCs, form the stem cell environment for neighboring GSCs, and CySCs have been proposed to be a source of instructive self-renewal signals^5^. CySCs provide the environment necessary to trigger GSC differentiation by the non-cell-autonomous approach^6^.

Early germ cells have been shown to be tightly controlled by niche signaling. Hub cells secrete unpaired (Upd) and hedgehog (Hh) proteins. Upd binds with Domeless (Dome) and activates the Janus kinase/signal transducer and the activator transcription (JAK/STAT) pathway in both GSCs and CySCs, and maintains their self-renewal ability^7,8^. Hh activates the Hh signaling pathway in CySCs, and is required for the maintenance of CySCs^9^. Two BMP-like molecules expressed in somatic cells, decapentaplegic (Dpp) and glass bottom boat (Gbb), are required for GSC maintenance and repress the differentiation factor bag-of-marbles (Bam) by bone morphogenetic protein (BMP) signaling^10^. Together with its regulator, benign gonial cell neoplasm (Bgcn), Bam is required for spermatogonia to transition from proliferation to differentiation^10–12^. Mutations in *bam* or *bgcn* result in germ cell tumors with extensive accumulation of undifferentiated germ cells^13,14^. Bam interacts with Bgcn and tumorous testis (Tut) to repress Mei-P26 expression, establishing a regulatory feedback loop that governs the proliferation of spermatogonia^15,16^.

*Drosophila* provides a simple system to investigate the complex genetic basis and related molecular mechanisms of biological events in reproduction^17–19^. Previously, a large-scale in vivo RNA interference (RNAi) screening in fly ovaries revealed the presence of a regulatory network involved in the self-renewal and differentiation of GSCs^20^. In the testis screen, Yu et al.^17^ found that protein synthesis and degradation, especially spliceosome and ribosome, were essential in the regulation of GSC homeostasis in fly testes.

CG5844 has been identified as a candidate GSC factor with its regulatory mechanism unclear. In this study, we named *CG5844* gene as *Spliceosome-Ribosome Linker Protein* (*Srlp*). Using in vivo and in vitro approaches, we systematically analyze the function and mechanism of Srlp in *Drosophila*. Here, we found that *Srlp* gene is essential for the self-renewal and differentiation of GSCs in *Drosophila* testis and increases proliferation and apoptosis in S2 cells. Moreover, Srlp regulates spliceosome and ribosome function via ribosomal protein L6 (RpL6) signals. In conclusion, the findings of this study will provide new insights into the mechanism underlying the stem cell niche.

## Results

### *Srlp* deficiency causes GSC self-renewal and differentiation defects

To determine the function of *Srlp* in *Drosophila* testes, we generated *Srlp* knockout flies using nos-cas9/CRISPR, resulting in a 335-bp deletion (264 bp in the coding sequence (CDS) region) and a code shift (Fig. S1a). The *Srlp* deletion in *Drosophila* was confirmed by PCR and sequencing (Fig. S1b and S1c). The homozygous *Srlp* mutation was lethal (*Srlp*^*–/–*^), while the heterozygous *Srlp* mutation (*Srlp*^*–/+*^) was viable and flies with the heterozygous mutation were fertile (Fig. S1d).

**Fig. 1:**
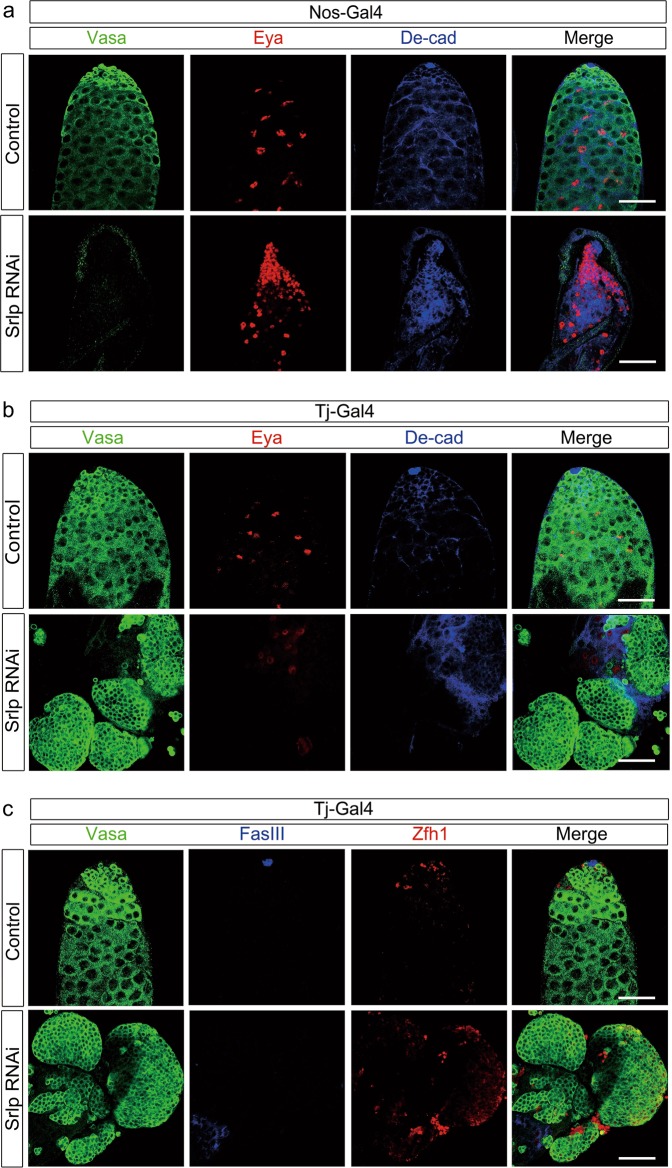
*Srlp* is required for the self-renewal and differentiation of germline stem cells (GSCs) in *Drosophila* testes. **a** Distribution of germ cells and somatic cells in the apex of the testis of control and nos>Srlp RNA interference (RNAi) flies. **b** Distribution of germ cells and somatic cells in the apex of the testis of control and tj>Srlp RNAi flies. **c** Distribution of germ cells and somatic cells in the apex of the testis of control and tj>Srlp RNAi flies. Immunostaining using anti-Vasa (green), anti-Eya (red in **a**, **b**), anti-DE-cad (blue in **a**, **b**), anti-Zfh1 (red in **c**), and anti-FasIII (blue in **c**) antibodies. Scale bar: 50 μm

To determine the in vivo function of *Srlp* in *Drosophila* testes, we generated a UAS/Gal4-mediated RNAi assay to test the loss of *Srlp* function using two different Gal4s (nos-Gal4 and tj-Gal4) that were mainly expressed in the stem cell niche^17^. Results of the immunofluorescence staining and confocal microscopic imaging of marker proteins revealed specific defects at the testicular apex. *Srlp* knockdown in early germ cells using nos-Gal4 caused tiny testes and complete absence of germ cells (cells were Vasa and TUNEL (terminal deoxynucleotidyl transferase-mediated dUTP-biotin nick-end labeling) negative), followed by accumulation of cyst cells (Fig. 1a and Fig. S2a). Moreover, *Srlp* knockdown driven by tj-Gal4 led to dysfunction of normal cyst cells and accumulation of undifferentiated germ cells, which ultimately developed into testis tumors (Fig. 1b, c), indicating that the *Srlp* gene is required for germ cell formation and cyst cell organization. Furthermore, these undifferentiated germ cells had proliferation and apoptosis ability (TUNEL and PH3 positive; Fig. S2b and S2c) without normal niche environments (Zfh1 positivity with abnormal pattern, no Eya-positive cyst cells, and no FasIII-positive hub cells; Fig. 1b, c).

### Srlp regulates proliferation and apoptosis in *Drosophila* S2 cells

**Fig. 2:**
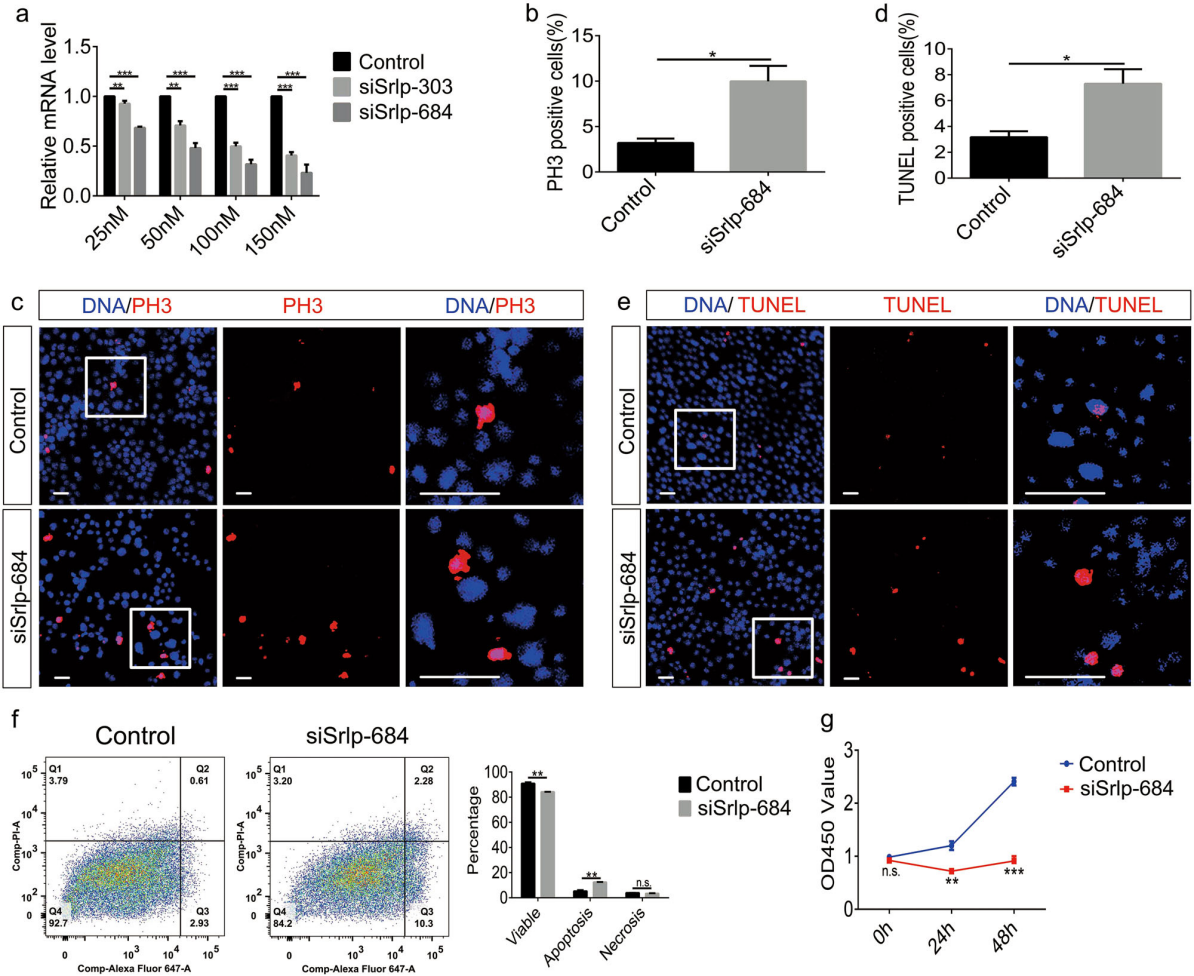
*Srlp* knockdown leads to proliferation and apoptosis in *Drosophila* S2 cells. **a** Relative *Srlp* messenger RNA (mRNA) level in negative control and siSrlp cells to validate knockdown efficiency. **b** Percentage of PH3-positive cells in control and siSrlp-684. **c** Immunostaining of control and siSrlp-684 S2 cells using anti-PH3 (red) and Hoechst-33342 (blue). **d** Percentage of TUNEL (terminal deoxynucleotidyl transferase-mediated dUTP-biotin nick-end labeling)-positive cells in control and siSrlp-684. **e** Immunostaining of control and siSrlp-684 S2 cells using TUNEL (red) and Hoechst-33342 (blue). **f** Flow cytometry testing of control and siSrlp-684. Ratio of viable cells decreased, and ratio of apoptosis cells increased. **g** Cell counting kit-8 (CCK-8) assay for control and siSrlp-684. Student’s *t*-test was used for the statistical analysis. *P *<* 0.05; **P *<* 0.01; ***P *<* 0.001; n.s. not significant. Error bars represent SEM. Scale bar: 30 μm

To further explore the function of Srlp in vitro, *Srlp* expression was silenced using two small interfering RNA (siRNAs; siSrlp-303 and siSrlp-684). Results of the quantitative reverse transcription-polymerase chain reaction (qRT-PCR) analysis revealed that siRNA suppression reduced the expression of *Srlp* messenger RNA (mRNA) in *Drosophila* S2 cells (Fig. 2a).

As down-regulation of *Srlp* expression caused defects in the self-renewal and differentiation of GSCs in *Drosophila*, we investigated whether *Srlp* was involved in proliferation and apoptosis. Importantly, both PH3-positive and TUNEL-positive cells significantly increased in siSrlp-684 (150 nM) S2 cells (Fig. 2b–e). Similar results were obtained by flow cytometry, reflecting an increased apoptosis ratio in cells following *Srlp* knockdown (Fig. 2f). Cell counting kit-8 (CCK-8 kit) was then used to detect whether the growth of S2 cells was affected by treatment with *Srlp* siRNA. The results indicated that knockdown of *Srlp* in S2 cells dramatically reduces cell growth (Fig. 2g).

**Fig. 3:**
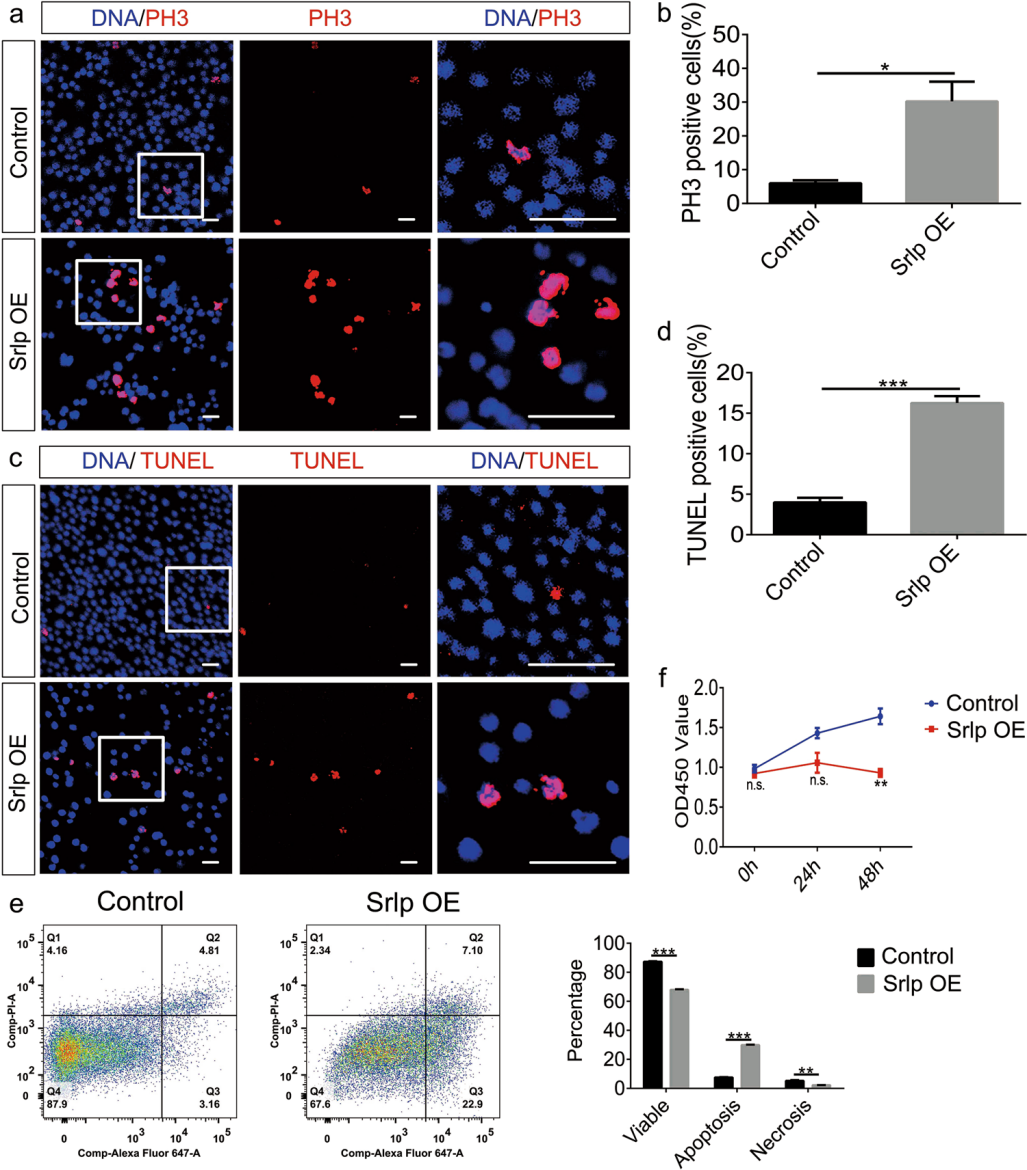
Srlp overexpression increases proliferation ability and cell death in *Drosophila* S2 cells. **a** Immunostaining of control and Srlp-overexpressing S2 cells using anti-PH3 (red) and Hoechst-33342 (blue). **b** Percentage of PH3-positive cells in the control and Srlp-overexpression group. **c** Immunostaining of the control and Srlp-overexpressing S2 cells by TUNEL (terminal deoxynucleotidyl transferase-mediated dUTP-biotin nick-end labeling) (red) and Hoechst-33342 (blue). **d** Percentage of TUNEL-positive cells in the control and Srlp-overexpression groups. **e** Flow cytometry of control and Srlp-overexpressing cells. The ratio of viable cells dramatically decreased while that of apoptosis cells significantly increased. **f** Cell counting kit-8 (CCK-8) assay of the control and Srlp-overexpressing S2 cells. Student’s *t*-test was used for the statistical analysis. *P *<* 0.05; **P *<* 0.01; ***P *<* 0.001; n.s. not significant. Error bars represent SEM. Scale bar: 30 μm

Next, we examined whether Srlp overexpression could lead to malfunctions in proliferation and apoptosis in S2 cells. We generated a UAS-Srlp-3xHA CDS clone driven by ub-Gal4. For the transient expression of Srlp, S2 cells were transfected with 0.2, 0.4, and 0.8 μg of plasmid separately. According to the results, the phenotype can only be observed when transfecting with 0.8 μg of plasmid (Fig. S3). Therefore, we chose 0.8 μg of plasmid for the transient expression of Srlp. Up-regulation of Srlp expression was confirmed at the transcription and translation levels (Fig. S4). Both PH3- and TUNEL-positive cells increased dramatically in S2 cells overexpressing Srlp (Fig. 3a–d), and their signals were even stronger than corresponding signals in siSrlp-684 (150 nM). Results of the flow cytometry showed that Srlp overexpression greatly reduced the ratio of viable cells and increased the apoptosis ratio (Fig. 3e). Results of the CCK-8 assay revealed that Srlp overexpression dramatically reduced the cell growth of S2 cells (Fig. 3f). Taken together, these results indicated that Srlp regulates cell proliferation and apoptosis.

### Srlp can rescue proliferation but not cell death

**Fig. 4:**
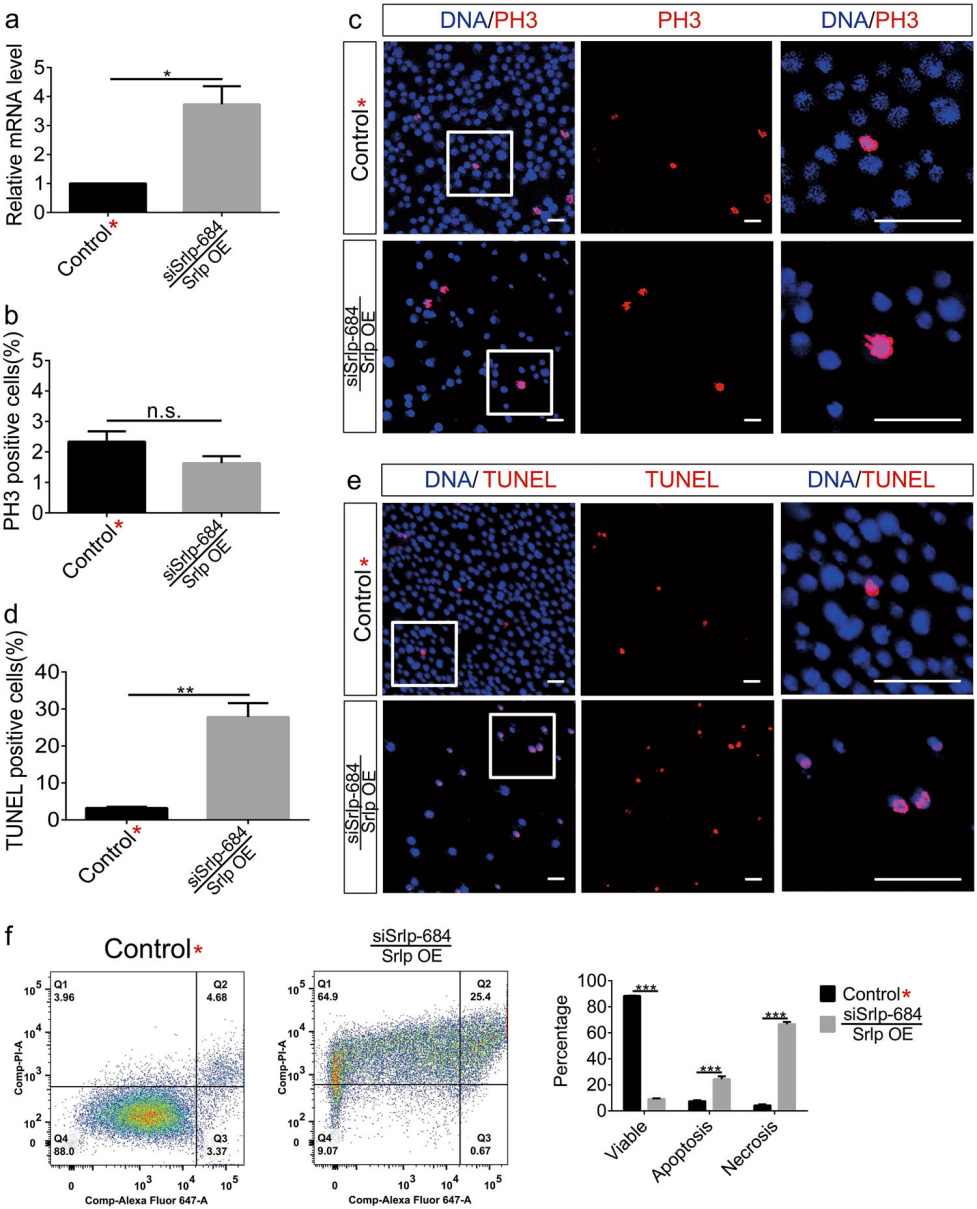
Rescue experiments of the *Srlp* knockdown phenotype. **a** Relative *Srlp* messenger RNA (mRNA) level of control* and siSrlp-684/Srlp-overexpressing S2 cells. **b** Percentage of PH3-positive cells in the control* and siSrlp-684/Srlp overexpression groups. **c** Immunostaining of the control* and siSrlp-684/Srlp-overexpressing S2 cells by anti-PH3 (red) and Hoechst-33342 (blue). **d** Percentage of TUNEL (terminal deoxynucleotidyl transferase-mediated dUTP-biotin nick-end labeling)-positive cells in control* and siSrlp-684/Srlp overexpression groups. **e** Immunostaining of control* and siSrlp-684/Srlp-overexpressing S2 cells using TUNEL (red) and Hoechst-33342 (blue). **f** Flow cytometry test of the control* and siSrlp-684/Srlp overexpression groups. The ratio of viable cells dramatically decreased while the ratios of apoptotic and necrotic cells significantly increased. Student’s *t*-test was used for the statistical analysis. *P *<* 0.05; **P *<* 0.01; ***P *<* 0.001; n.s. not significant. Error bars represent SEM. Scale bar: 30 μm

We conducted two independent Srlp rescue assays in S2 cells to further confirm their phenotype. Results of the qRT-PCR analysis indicated that Srlp overexpression recovered the *Srlp* mRNA expression of siSrlp-684 (Fig. 4a). Immunofluorescence staining showed that cell proliferation ability (indicated by PH3 positivity) could be rescued by Srlp overexpression (Fig. 4b, c). However, the TUNEL-positive cell ratio of *Srlp* siRNA could not be rescued by Srlp overexpression; on the contrary, it was enhanced by overexpression, reflecting a superposition effect for cell survival defects (Fig. 4d, e). Component analysis of cell death by flow cytometry showed that Srlp overexpression in cells silenced by siSrlp-684 reduced the number of viable cells and dramatically increased the numbers of apoptotic and necrotic cells (Fig. 4f). Interestingly, *Srlp* knockdown could partially rescue the *Srlp* expression of overexpressed Srlp, and similar results were observed in the Srlp overexpression/siSrlp-684 rescue assay (Fig. S5). Taken together, our data indicated that Srlp rescue assays restored the proliferation ability but accelerated cell death.

### Ribosomes were enriched in the regulatory network of Srlp-binding proteins

**Fig. 5:**
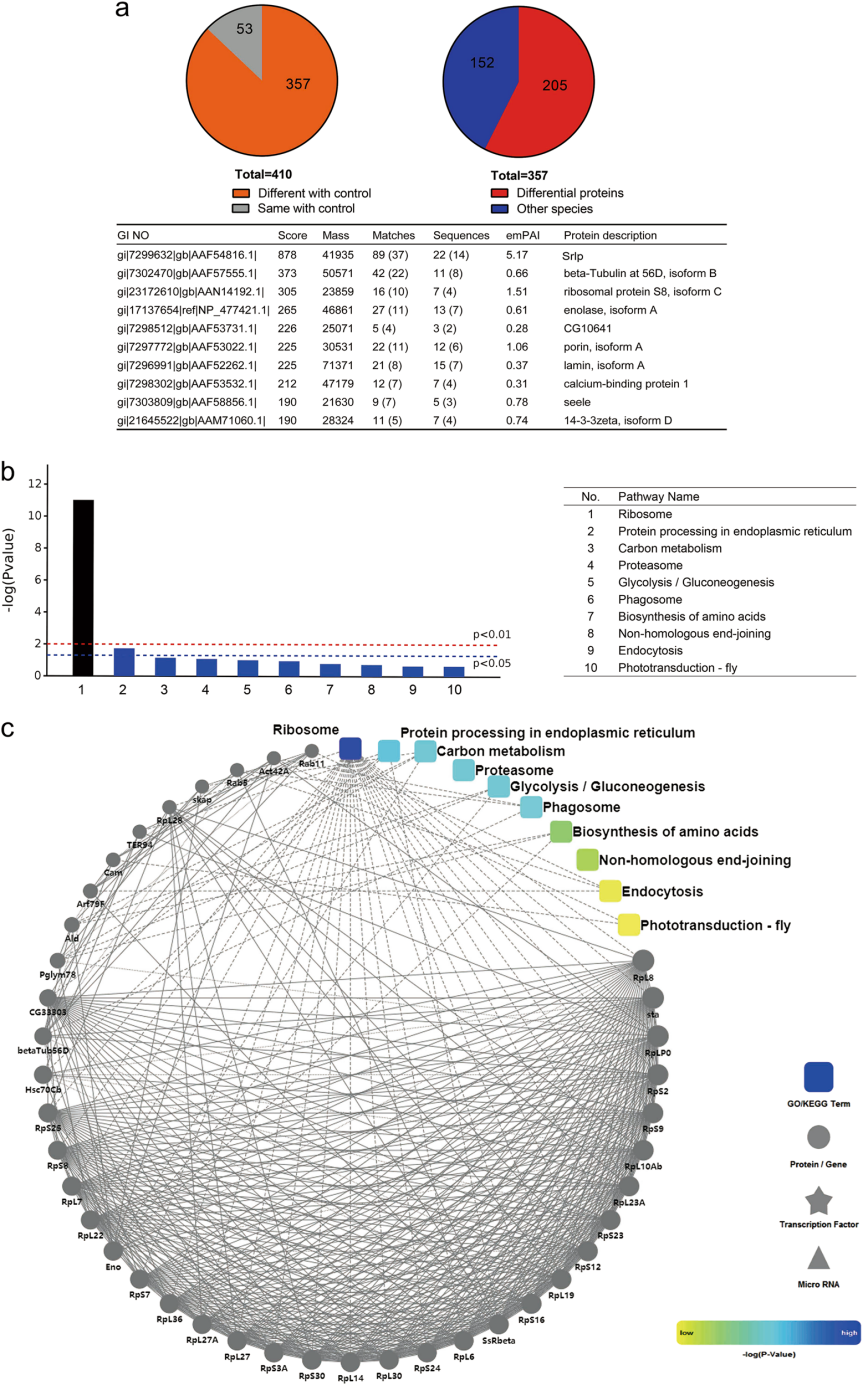
Characteristics of Srlp-binding proteins in *Drosophila* melanogaster. **a** Identification of Srlp-binding proteins. **b** KEGG (Kyoto Encyclopedia of Genes and Genomes) pathway analysis of identified Srlp-binding proteins in *Drosophila* S2 cells. **c** Protein-protein interaction (PPI) analysis of identified Srlp-binding proteins from KEGG pathway analysis in *Drosophila* S2 cells. Dots represent genes/proteins, and rounded rectangles represent biological processes, cell localization, molecular functions or signaling pathways. Solid lines represent verified interactions, and dashed lines represent predicted interactions

To explore the regulatory network of Srlp-binding proteins, we pulled down Srlp-HA fusion proteins with HA beads and identified Srlp-related binding proteins by liquid chromatography-tandem mass spectrometry (LC-MS/MS). Thereafter, we identified 205 Srlp-binding proteins in *Drosophila* melanogaster (Fig. 5a and Table S1). The ten Srlp-binding proteins with the highest scores are listed in Fig. 5a. Bioinformatics analysis was performed by the OmicsBean data integration analysis platform, and classified according to the biological process, cellular component, and molecular function categories. Figure S6 shows the top 20 enriched and significantly different terms based on gene ontology (GO) analysis. In the biological process category, translation, biosynthetic and metabolic processes, and mitotic spindle elongation were mainly enriched in Srlp-binding proteins. In the context of cellular component, cytoplasm, ribosome, and ribonucleoprotein complex were involved in Srlp-binding proteins. Moreover, in the molecular function category, Srlp was found to be related with the structural constituent of ribosome, structural molecular activity, isomerase activity, and RNA binding. Enrichment analysis using the KEGG (Kyoto Encyclopedia of Genes and Genomes) pathway and protein–protein interaction (PPI) demonstrated that the identified Srlp-binding proteins were involved in the ribosome signaling pathway with high confidence (*p* < 0.01), indicating the significant roles of crosstalk between Srlp and the ribosome complex (Fig. 5b, c).

### Srlp regulated the expression levels of spliceosome and ribosome subunits

**Fig. 6:**
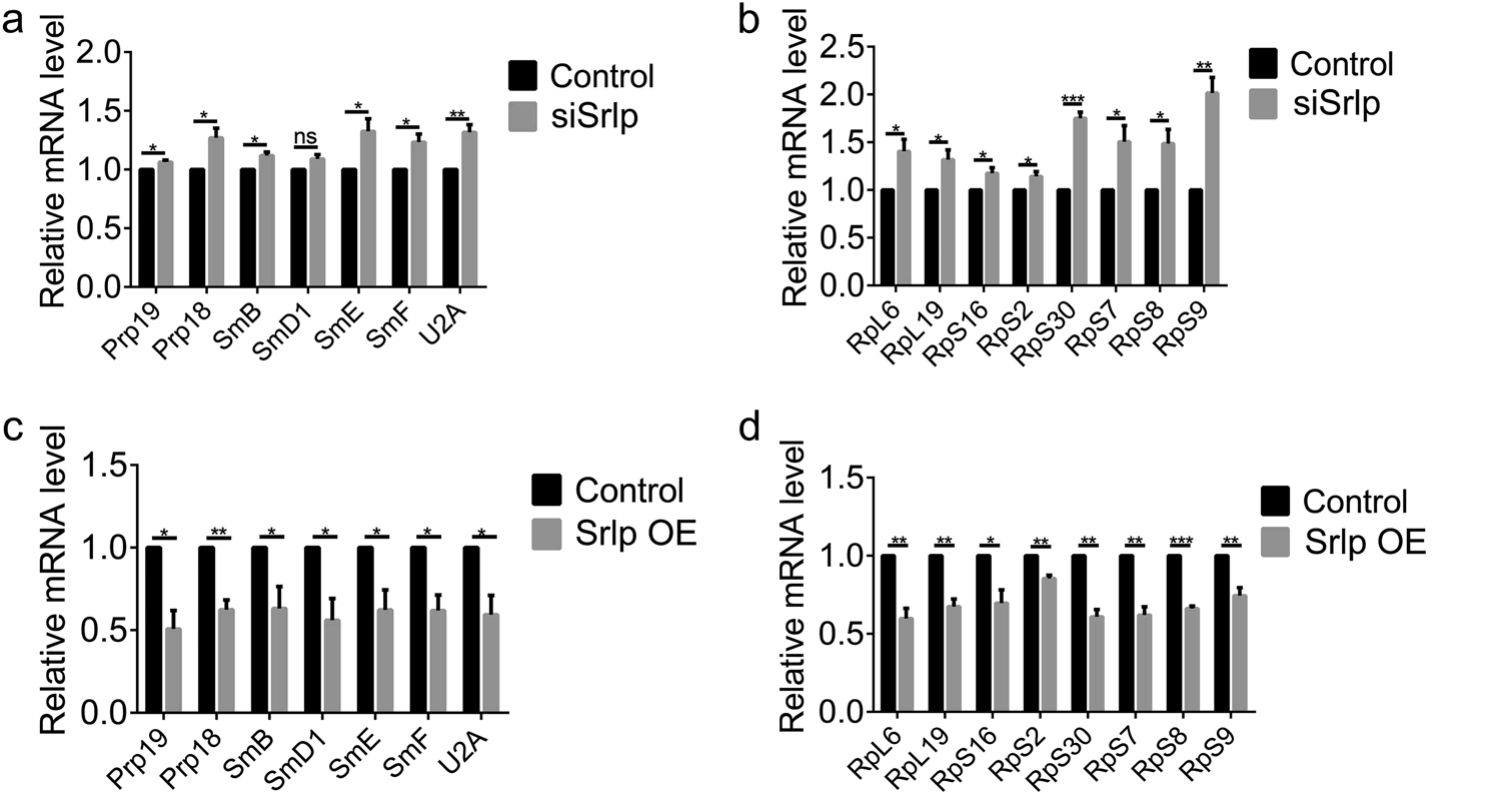
Srlp regulates the expression level of major spliceosome and ribosome subunits. **a** Relative messenger RNA (mRNA) level of spliceosome subunits (*Prp18*, *Prp19*, *SmB*, *SmD1*, *SmE*, *SmF*, and *U2A*) in the control and siSrlp-684 S2 cells. **b** Relative mRNA levels of ribosome subunits (*RpL19*, *RpS2*, *RpS7*, *RpS8*, *RpS9*, *RpS16*, and *RpS30*) in control and siSrlp-684 S2 cells. **c** Relative mRNA level of spliceosome subunits (*Prp18*, *Prp19*, *SmB*, *SmD1*, *SmE*, *SmF*, and *U2A*) in control and Srlp-overexpressing S2 cells. **d** Relative mRNA level of ribosome subunits (*RpL19*, *RpS2*, *RpS7*, *RpS8*, *RpS9*, *RpS16*, and *RpS30*) in control and Srlp-overexpressing S2 cells. Student’s *t*-test was used. *P *<* 0.05; **P *<* 0.01; ***P *<* 0.001; n.s. not significant. Error bars represent SEM

Many proteins involved in protein synthesis and degradation were identified in the screening of proteins that bound to Srlp. To further investigate whether Srlp affected the function of spliceosome and ribosome, we measured the expression level of major spliceosome and ribosome subunits. Spliceosome subunits (*Prp18*, *Prp19*, *SmB*, *SmD1*, *SmE*, *SmF*, and *U2A*) and ribosome subunits (*RpL6*, *RpL19*, *RpS2*, *RpS7*, *RpS8*, *RpS9*, *RpS16*, and *RpS30*) were all up-regulated in siSrlp S2 cells and down-regulated in S2 cells overexpressing Srlp (Fig. 6), indicating that Srlp competitively controlled the expression level of spliceosome and ribosome, which may affect their function.

### Srlp regulates ribosome function via RpL6 in *Drosophila*

**Fig. 7:**
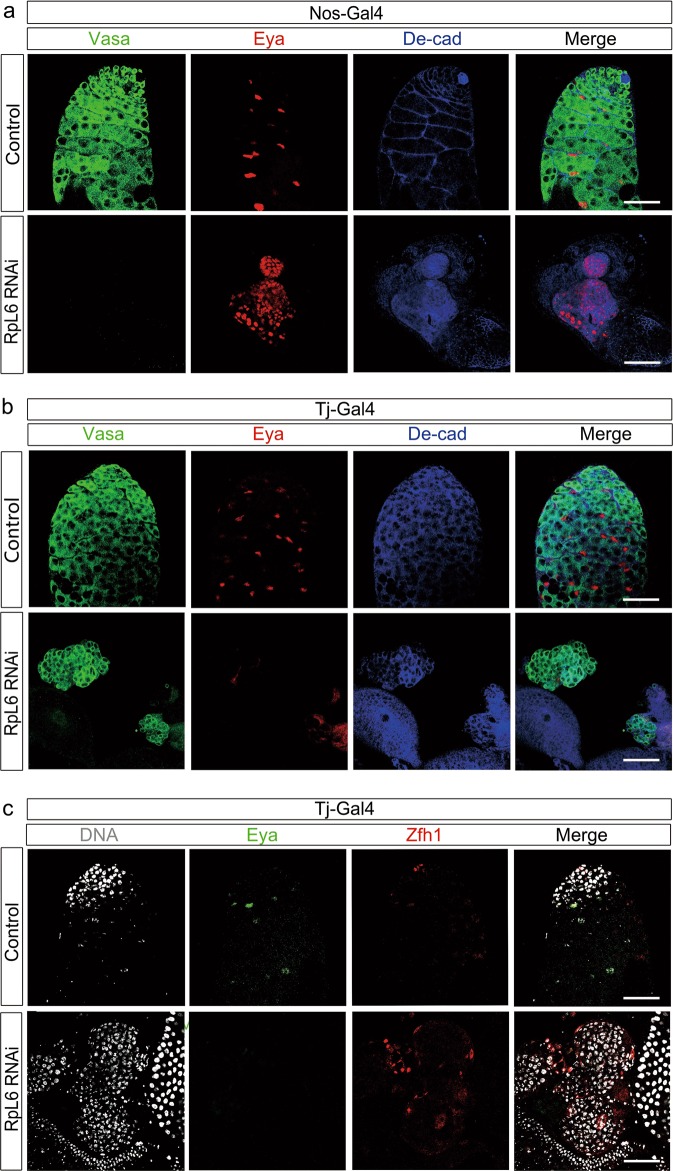
Knockdown of *RpL6* by nos-Gal4 and tj-Gal4 causes tiny testes and germ cell dysfunction. **a** Distribution of germ cells and somatic cells in the apex of the testis in the control and nos>RpL6 RNA interference (RNAi) flies. **b** Distribution of germ cells and somatic cells in the apex of the testis in the control and tj>RpL6 RNAi flies. **c** Distribution of germ cells and somatic cells in the apex of testis in control and tj>RpL6 RNAi flies. Immunostaining using anti-Vasa (green in **a**, **b**), anti-Eya (red in **a**, **b**; green in **c**), anti-DE-cad (blue in **a**, **b**), anti-Zfh1 (red in **c**), and Hoechst-33342 (gray in **c**). Scale bar: 50 μm

To elucidate the potential mechanism of Srlp, we investigated whether ribosome subunits such as RpL6 were essential for self-renewal and differentiation of cells in *Drosophila* testes. Interestingly, *RpL6* knockdown in early germ cells driven by nos-Gal4 caused tiny testes, loss of germ cells, and accumulation of cyst cells (Fig. 7a). Knockdown of *RpL6* in cyst cells by tj-Gal4 led to accumulation of undifferentiated germ cells and subsequent tumor formation (Fig. 7b, c). These GSC-like germ cells exhibited the ability for self-renewal without normal maintenance of the apical hubs and CySCs (Zfh1-positive and DE-cad-positive cells existed in an abnormal pattern, and there were no Eya-positive cyst cells). Our results indicated that the *RpL6* gene completely mimicked the self-renewal and differentiation phenotype of the *Srlp* gene in GSCs in *Drosophila* testes.

Significantly, spliceosome subunits (*Prp18*, *Prp19*, *SmB*, *SmD1*, *SmE, SmF*, and *U2A*) and ribosome subunits (*RpL19*, *RpS2*, *RpS7*, *RpS8*, *RpS9*, *RpS16*, and *RpS30*) were all down-regulated in S2 cells overexpressing RpL6 (Fig. S7). Additionally, RpL6-overexpressing S2 cells exhibited proliferation ability (Fig. S8 a–d), and dramatically promoted the cell death process, as evidenced by increased ratios of apoptotic and necrotic cells (Fig. S8 e–g).

**Fig. 8:**
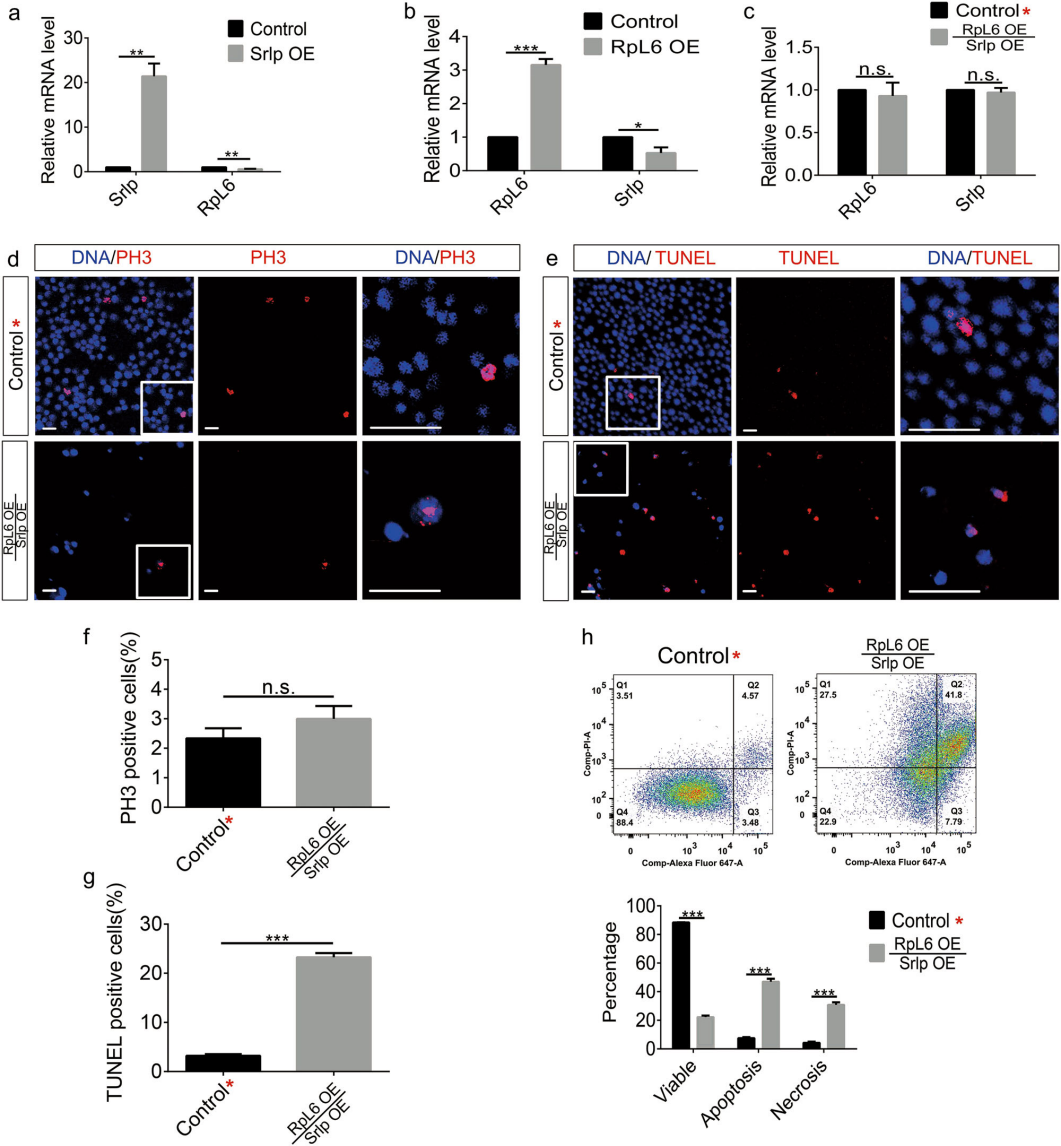
Effects of rescue of S2 cells with the RpL6 overexpression phenotype by Srlp overexpression. **a** Relative *Srlp* and *RpL6* messenger RNA (mRNA) levels in the control and *Srlp*-overexpressing S2 cells. **b** Relative *RpL6* and *Srlp* mRNA levels in the control and RpL6-overexpressing S2 cells. **c** Relative *RpL6* and *Srlp* mRNA levels in the control* and RpL6-overexpressing/Srlp-overexpressing S2 cells. **d** Immunostaining of the control* and RpL6-overexpressing/Srlp-overexpressing S2 cells using anti-PH3 (red) and Hoechst-33342 (blue). **e** Immunostaining of the control* and RpL6-overexpressing/Srlp-overexpressing S2 cells using TUNEL (terminal deoxynucleotidyl transferase-mediated dUTP-biotin nick-end labeling) (red) and Hoechst-33342 (blue). **f** Percentage of PH3-positive cells in the control* and RpL6-overexpressing/Srlp-overexpressing S2 cells. **g** Percentage of TUNEL-positive cells in the control* and RpL6-overexpressing/Srlp-overexpressing S2 cells. **h** Flow cytometry test of the control* and RpL6-overexpressing /Srlp-overexpressing S2 cells. The ratio of viable cells dramatically decreased and the ratio of apoptotic and necrotic cells significantly increased. Student’s *t*-test was used for statistical analysis. *P *<* 0.05; **P *<* 0.01; ***P *<* 0.001; n.s. not significant. Error bars represent SEM. Scale bar: 30 μm

Next, cells overexpressing Srlp alone inhibited *RpL6* expression, while cells overexpressing RpL6 alone repressed *Srlp* expression (Fig. 8a, b). As it was obvious that *RpL6* and *Srlp* were mutually antagonistic, we further tested whether overexpression of both RpL6 and Srlp could restore the proliferation ability and apoptosis in S2 cells. First, *Srlp* and *RpL6* expression levels could be recovered in RpL6 overexpression/Srlp overexpression or Srlp overexpression/RpL6 overexpression S2 cells (Fig. 8c and Fig. S9a). Second, in cells overexpressing RpL6, the increased proliferation ability, but not the ratios of apoptotic and necrotic cells, could be partially rescued by Srlp in S2 cells (Fig. 8d–h). Furthermore, the increased proliferation in Srlp-overexpressing cells could be partially rescued by RpL6, while the ratios of apoptosis and necrosis (especially the necrosis ratio) dramatically increased (Fig. S9 b–f).

Co-transfecting S2 cells with both Srlp and RpL6 increased the expression level of *Srlp* and drastically suppressed the expression level of *RpL6* (Fig. S10a and S10b). Results of the co-immunoprecipitation assay confirmed that the Srlp protein could combine with the RpL6 protein, which is consistent with LC-MS/MS data (Fig. S10c). Together, our results illustrate that RpL6 is a Srlp-binding protein and a potential target of Srlp. These results indicate that Srlp regulates the proliferation ability and cell survival via RpL6 in *Drosophila* testis cells.

## Discussion

The stem cell niche controls normal self-renewal and differentiation of GSCs^17,21^. Srlp was identified as a regulatory factor of GSCs in *Drosophila* testes; however, its mechanism remains to be elucidated. This is the first study to investigate the function and mechanism of Srlp in *Drosophila* in vivo and in vitro. In this study, we used *Drosophila* as a model and found that Srlp is essential for the self-renewal and differentiation of GSCs in the testis and increases the proliferation and apoptosis of S2 cells. The phenotype of proliferation but not apoptosis, which was induced by silencing the *Srlp* gene, could be rescued by Srlp overexpression in S2 cells. Identification of Srlp-binding proteins by LC-MS/MS indicated that ribosome was enriched. Importantly, Srlp could affect the expression pattern of splicesome and ribosome subunits by competitive integration. Overexpressed RpL6 decreased the expression levels of *Srlp*, splicesome subunits, and ribosome subunits. This study demonstrated that Srlp regulated splicesome and ribosome function via RpL6 signals.

Previous studies have demonstrated that the regulation of protein synthesis is crucial for self-renewal and differentiation of GSCs in both fly testes and ovaries^17,22–26^. In the testis screen, nine ribosome proteins, four spliceosome-associated proteins, and three eIF3 complex proteins contribute to both GSC maintenance and early germ cell differentiation^17^. The switch from TA proliferation to differentiation in the testis is mediated by translational control of Mei-P26 and differentiation factor Bam^15,16,27^. Mei-P26 is a key molecule regulated by the translational machinery that controls distinct developmental programs during germ cell formation and maturation^27,28^. In UAS-Srlp RNAi and UAS-RpL6 RNAi testes driven by tj-Gal4, overproliferation of undifferentiated germ cells accumulate in the testes, consequently leading to tumor formation. These GSC-like cells acquired proliferation and apoptosis abilities and were devoid of distinguishable hub cells, indicating that the overproliferated GSC-like cells are self-sustained.

Spliceosome and ribosome are of great importance in the occurrence of diseases^29,30^. The major spliceosome is essential for mRNA processing and cell survival^31^. Mutation of U2A, a major spliceosome subunit, impaired the differentiation of spermatogonia, abolishing the maturation of germ cells into sperm^18^. Zfrp8 functions in the formation of mRNA ribonucleoprotein (RNP) complexes, and is essential for the maintenance of follicles and GSCs in the ovary^32^. Zfrp8 functions in the late stages of ribosome assembly and may regulate the binding of specific mRNA-RNPs to the small ribosomal subunit (RpS2), ultimately controlling their cytoplasmic localization and translation^33^. Previously, a novel class of RNPs, termed regulatory RNPs, has been found to contribute to the biogenesis of small nuclear RNPs and ribosome heterogeneity^34^. Specifically, the results of our study revealed that Srlp regulates splicesome and ribosome function via RpL6 signals. These findings suggest that Srlp may affect spliceosome and ribosome assembly, ultimately leading to loss of control of cell fate determination.

Previous studies have reported that apoptosis can promote the proliferation ability and the phenomenon of apoptosis-induced proliferation existed in many cell lines^35,36^. Moreover, apoptotic cells also had active roles for proliferation in *Drosophila*^37,38^. *Srlp* knockdown or Srlp-overexpressing S2 cells had a significantly higher proportion of PH3-positive and TUNEL-positive cells, indicating that Srlp is vital for the homeostasis of proliferation and apoptosis processes.

In summary, the present study mainly discussed the crosstalk between Srlp and large ribosomal subunit RpL6. Our findings demonstrated that Srlp, together with RpL6, regulated GSC self-renewal and differentiation by controlling spliceosome and ribosome function in *Drosophila* testis. This study may provide new insights into the pathogenic mechanism of male sterility and testicular germ cell tumor.

## Materials and methods

### Fly strains and RNAi strategy

All flies were cultured on standard corn meal food at 25 °C. The transgenic RNAi flies used in the screen were ordered from the Tsinghua Fly Center (THFC) and were from the same RNAi collection as the TRiP (Transgenic RNAi Project). Information for alleles and transgenes used in this study can be found either in FlyBase or as noted: Nos-Gal4 (BDSC, 4937), Tj-Gal4 (DGRC, 104055), UAS-Srlp RNAi (THFC, THU1733), UAS-RpL6 RNAi (THFC, THU1350).

The crosses were set and raised at room temperature (25 °C). Male Gal4 drivers were crossed to the transgenic UAS-RNAi virgin females. The male progenies were dissected for further function analysis.

### Plasmid construction

Srlp CDS (a gift from C. Tong) was subcloned into the pUAS-attB-3xHA vector, and the CDS sequence was amplified by PCR using primers to introduce *Not*I (1166A, Takara, Japan) and *Xba*I (1093A, Takara, Japan) restriction sites. RpL6 CDS (a gift from C. Tong) was subcloned into the pUAS-attB vector, and the sequence was amplified by PCR using primers to introduce a V5 tag as well as *Not*I and *Xba*I restriction sites. Plasmid construction protocol by classical restriction ligation cloning has been described before^39^. Following primers were used: pUAS-attB-3xHA-Srlp F: 5-ATAAGAATGCGGCCGCGATGCTTCGCAAATTTGGAGGTC-3, R: 5-GCTCTAGACTACTTTTCGTTTGGCTTATCCTTGG-3; pUAS-attB-RpL6-V5 F: 5-ATAAGAATGCGGCCGCATGGCACCCATCGAGAAAGC-3, R: 5-GCTCTAGATTACGTAGAATCGAGACCGAGGAGAGGGTTAGGGATAGGCTTACCGAATCGCATGCGGTGGGGGTAT-3; pUAS-attB seq primer: CCAGCAACCAAGTAAATCAA.

### Cell culture and transfection

*Drosophila* Schneider 2 (S2) cells were obtained from *Drosophila* Genomics Resource Center and were grown at 28 °C in Schneider’s *Drosophila* medium (21720024, Gibco, USA) supplemented with 10% heat-inactivated fetal bovine serum (04-001-1ACS, Bioind, Israel). The cells were split with supplemented medium at a ratio of 1:4 every 3–4 days which has been described before^40^.

S2 cells were plated in the wells of six-well plates 1 day before transfection. For knockdown of *Srlp*, S2 cells were transfected using Lipofectamine 2000 Transfection Reagent (Lipo2000; 11668019, Invitrogen, USA). Two tubes, one containing siRNA with 250 μL of opti-Minimal Essential Medium (MEM) (31985-062, Gibco, USA) and another containing a corresponding dose of Lipo2000 with 250 μL of opti-MEM, were mixed and incubated for 5 min at room temperature. Then, the tubes were mixed and incubated for 20 min at room temperature. The siRNAs were designed and synthesized by GenePharma company (Suzhou, China). The siRNA information is described as follows: negative control F: 5-UUCUCCGAACGUGUCACGUTT-3, R: 5-ACGUGACACGUUCGGAGAATT-3; siSrlp-303 F: 5-GCUUACUGCAUCGCAGCUUTT-3, R: 5-AAGCUGCGAUGCAGUAAGCTT-3; siSrlp-684 F: 5-GCGUGCCCUGGACCUGAUATT-3, R: 5-UAUCAGGUCCAGGGCACGCTT-3.

For transient expression of Srlp and RpL6, S2 cells were transfected with plasmid using Effectene Transfection Reagent (301425, Qiagen, Germany). Briefly, plasmids were diluted to 100 μL with buffer EC and 6.4 μL Enhancer, vortexed, and incubated for 5 min at room temperature, To this, 20 μL of Effectene Transfection Reagent was added for an additional 10 min, to which 600 μL of opti-MEM was mixed, and the mixture was used as the final culture medium.

The detailed plasmid and culture times are as follows:

(1) Control (negative control or ub-Gal4+pUAS-attB): culture for 48 h.

(2) siSrlp (siRNA): culture for 48 h.

(3) Srlp OE (ub-Gal4+pUAS-attB-3xHA-Srlp): culture for 48 h.

(4) RpL6 OE (ub-Gal4+pUAS-attB-RpL6-V5): culture for 48 h.

(5) Srlp OE+RpL6 OE (ub-Gal4+pUAS-attB-3xHA-Srlp+pUAS-attB-RpL6-V5): culture for 48 h.

(6) Control*: (a) negative control culture for 48 h, followed by culture with ub-Gal4+pUAS-attB for 48 h; (b) ub-Gal4+pUAS-attB culture for 48 h, followed by negative control culture for 48 h; and (c) ub-Gal4+pUAS-attB culture for 96 h (corresponding to the experimental group).

(7) siSrlp/Srlp OE: siSrlp culture for 48 h, followed by culture of Srlp OE for 48 h.

(8) Srlp OE/ siSrlp: culture of Srlp OE cells for 48 h, followed by culture of siSrlp cells for 48 h.

(9) Srlp OE/RpL6 OE: Srlp OE culture for 48 h, followed by culture of RpL6-overexpressing cells for 48 h.

(10) RpL6 OE/Srlp OE: RpL6 OE culture for 48 h, followed by Srlp OE culture for 48 h.

### Quantitative reverse transcription-PCR

Total RNA was extracted using Trizol reagent (9108, Takara, Japan). Complementary DNA was synthesized using Prime Script RT Reagent Kit (RR037A, Taraka, Japan), and qRT-PCR was performed using SYBR Premix Ex Taq (RR420A, Takara, Japan). Glyceraldehyde 3-phosphate dehydrogenase (GAPDH) was amplified as an internal standard. Fold changes were calculated using the standard curve according to the manufacturer’s protocol. Each experiment was independently repeated three times. All primers used for qRT-PCR are listed in Table S2.

### Immunofluorescence and antibodies

Fly testes were dissected in 1× phosphate-buffered saline (PBS) and fixed for 30 min in 4% paraformaldehyde. After washing three times in 1× PBS with 0.1% Triton X–100 (PBST) and blocking for 1 h in 5% bovine serum albumin, the samples were incubated with primary antibodies overnight at 4 °C. After washing three times for 10 min in 0.1% PBST, the samples were incubated for 1 h with secondary antibodies at room temperature followed by three times washing in 0.1% PBST. Testes were then stained with Hoechst-33342 (1.0 mg/mL, Invitrogen) for 5 min before mounting. Images were captured on an LSM710 Zeiss confocal microscope and processed using Adobe Photoshop CS5 software. *Drosophila* S2 cells were cultured for 24 h, and immunostaining was carried out in the culture dish according to the protocols described above.

The antibodies used were as follows: mouse anti-Eya (Developmental Studies Hybridoma Bank, 1:20); rat anti-DE-cadherin (Developmental Studies Hybridoma Bank, 1:20); mouse anti-FasIII (Developmental Studies Hybridoma Bank, 1:50); rabbit anti-Vasa (a gift from C. Tong, 1:1000); rat anti-Zfh1 (a gift from C. Tong, 1:2000); rabbit anti-PH3 (53348, Cell Signaling Technology, 1:1000); anti-V5 (R960-25, Invitrogen, 1:500); and rabbit anti-HA (3724, Cell Signaling Technology, 1:1000). Secondary antibodies conjugated to A488, Cy3, A594, or A647 (Molecular Probes and Jackson Immunologicals) were diluted at 1:1000.

### TUNEL assay

Cell apoptosis was determined using the TUNEL assay according to the manufacturer’s protocols. The TUNEL BrightRed Apoptosis Detection Kit was obtained from Vazyme (A113, Nanjing, China).

### Flow cytometry assay

Flow cytometry was performed using an Annexin V-Alexa Fluor 647/propidium iodide (PI) Apoptosis Assay Kit (FMSAV647-100, FcMACS, Nanjing, China). After transfection for 48 h, S2 cells were washed with ice-cold PBS. Different cell groups were stained with the apoptosis detection kit according to the manufacturer’s instructions. Cells (1 × 10^6^ cells per well) from each sample were suspended and incubated in 250 μL of binding buffer. Then, 100 µL of the cell suspension was incubated with 5 μL of Annexin V-Alexa Fluor 647 and 10 μL of PI for 15 min at room temperature in the dark. Then, 200 μL 1× PBS was added into each sample and mixed. The samples were then analyzed using FACScan flow cytometry (BD Biosciences, San Jose, CA, USA). Data were calculated at different intervals. Flow cytometry assay was performed with at least three independent experiments.

### Cell viability assay

CCK-8 assay (CK04-3000T, DOJINDO, Japan) was utilized to assess *Drosophila* S2 cell viability according to the manufacturer’s protocols. Briefly, transfected S2 cell were transferred to 96-well plates (3000 cells per well), and incubated in 10% CCK-8 reagent that was diluted in Schneider’s *Drosophila* medium at 37 °C for 1 h. After transfection at 0, 24, and 48 h, the absorbance in each well was evaluated at 450 nm (Multiskan GO, Thermo Scientific, Waltham, USA). All experiments were repeated at least three times.

### LC-MS/MS

Protein extraction and digestion were performed according to the methods described before^41^. The protein digests were separated using a 10 min elution gradient at a flow rate of 2 µL per min in an Eksigent nanoLC-Ultra 2D system (AB SCIEX). A C18 reversed phase chromatographic column (75 μm × 15 cm, 3 μm, 120 Å, ChromXP Eksigent) was used as the analytical column. MS/MS scan was performed by tripleTOF5600 system (AB SCIEX).

### Bioinformatics analysis

HA-Srlp protein in control and Srlp-overexpressing S2 cells are pulled down by HA beads, and followed by LC-MS/MS identification. *Drosophila* melanogaster proteins identified in Srlp-overexpression group and not in control group were considered as candidates of Srlp-binding proteins. To analyze the functional characteristics of Srlp-binding proteins, the OmicsBean data integration analysis platform was used to perform GO functional annotation, KEGG Pathway, and PPI analysis.

### Statistical analysis

Experiments were repeated at least three times. The quantitative results are presented as mean ± standard error of mean (SEM). The data were evaluated for statistical differences using Student’s *t*-test and one-way analysis of variance by Graphpad Software (https://www.graphpad.com/) with *P < 0.05; **P < 0.01; ***P < 0.001.

## Supplementary information

Supplementary materials and methods

Figure S1

Figure S2

Figure S3

Figure S4

Figure S5

Figure S6

Figure S7

Figure S8

Figure S9

Figure S10

Table S1

Table S2

Supplementary figure legends

## References

[1] Davies EL, Fuller MT (2008). Regulation of self-renewal and differentiation in adult stem cell lineages: lessons from the *Drosophila* male germ line. Cold Spring Harb. Symp. Quant. Biol..

[2] de Cuevas M, Matunis EL (2011). The stem cell niche: lessons from the *Drosophila* testis. Development.

[3] Losick VP, Morris LX, Fox DT, Spradling A (2011). *Drosophila* stem cell niches: a decade of discovery suggests a unified view of stem cell regulation. Dev. Cell.

[4] Carbonell A, Pérez-Montero S, Climent-Cantó P, Reina O, Azorín F (2017). The germline linker histone dBigH1 and the translational regulator Bam form a repressor loop essential for male germ stem cell differentiation. Cell Rep..

[5] Leatherman JL, Dinardo S (2010). Germline self-renewal requires cyst stem cells and stat regulates niche adhesion in *Drosophila* testes. Nat. Cell Biol..

[6] Lim JG, Fuller MT (2012). Somatic cell lineage is required for differentiation and not maintenance of germline stem cells in *Drosophila* testes. Proc. Natl. Acad. Sci. USA.

[7] Kiger AA, Jones DL, Schulz C, Rogers MB, Fuller MT (2001). Stem cell self-renewal specified by JAK-STAT activation in response to a support cell cue. Science.

[8] Tulina N, Matunis E (2001). Control of stem cell self-renewal in *Drosophila* spermatogenesis by JAK-STAT signaling. Science.

[9] Zhang Z, Lv X, Jiang J, Zhang L, Zhao Y (2013). Dual roles of Hh signaling in the regulation of somatic stem cell self-renewal and germline stem cell maintenance in *Drosophila* testis. Cell Res..

[10] Kawase E, Wong MD, Ding BC, Xie T (2004). Gbb/Bmp signaling is essential for maintaining germline stem cells and for repressing bam transcription in the *Drosophila* testis. Development.

[11] Bunt SM, Hime GR (2004). Ectopic activation of Dpp signalling in the male *Drosophila* germline inhibits germ cell differentiation. Genesis.

[12] Shivdasani AA, Ingham PW (2003). Regulation of stem cell maintenance and transit amplifying cell proliferation by tgf-beta signaling in *Drosophila* spermatogenesis. Curr. Biol..

[13] Eun SH (2013). MicroRNAs downregulate Bag of marbles to ensure proper terminal differentiation in the *Drosophila* male germline. Development.

[14] Gönczy P, Matunis E, DiNardo S (1997). bag-of-marbles and benign gonial cell neoplasm act in the germline to restrict proliferation during *Drosophila* spermatogenesis. Development.

[15] Chen D (2014). Three RNA binding proteins form a complex to promote differentiation of germline stem cell lineage in *Drosophila*. PLoS Genet..

[16] Insco ML (2012). A self-limiting switch based on translational control regulates the transition from proliferation to differentiation in an adult stem cell lineage. Cell Stem Cell.

[17] Yu J (2016). Protein synthesis and degradation are essential to regulate germline stem cell homeostasis in *Drosophila* testes. Development.

[18] Wu H (2016). Major spliceosome defects cause male infertility and are associated with nonobstructive azoospermia in humans. Proc. Natl. Acad. Sci. USA.

[19] Yu J (2015). Identification of seven genes essential for male fertility through a genome-wide association study of non-obstructive azoospermia and RNA interference-mediated large-scale functional screening in *Drosophila*. Hum. Mol. Genet..

[20] Yan D (2014). A regulatory network of *Drosophila* germline stem cell self-renewal. Dev. Cell.

[21] Xie T (2013). Control of germline stem cell self-renewal and differentiation in the *Drosophila* ovary: concerted actions of niche signals and intrinsic factors. Wiley Interdiscip. Rev. Dev. Biol..

[22] Sanchez CG (2016). Regulation of ribosome biogenesis and protein synthesis controls germline stem cell differentiation. Cell Stem Cell.

[23] Lee JY, Chen JY, Shaw JL, Chang KT (2016). Maintenance of stem cell niche integrity by a novel activator of integrin signaling. PLoS Genet..

[24] Shen R, Weng C, Yu J, Xie T (2009). eIF4A controls germline stem cell self-renewal by directly inhibiting BAM function in the *Drosophila* ovary. Proc. Natl. Acad. Sci. USA.

[25] Spradling A, Fuller MT, Braun RE, Yoshida S (2011). Germline stem cells. Cold Spring Harb. Perspect. Biol..

[26] Wang Z, Lin H (2004). Nanos maintains germline stem cell self-renewal by preventing differentiation. Science.

[27] Li Y, Maines JZ, Tastan OY, McKearin DM, Buszczak M (2012). Mei-P26 regulates the maintenance of ovarian germline stem cells by promoting BMP signaling. Development.

[28] Neumüller RA (2008). Mei-P26 regulates microRNAs and cell growth in the *Drosophila* ovarian stem cell lineage. Nature.

[29] Singh RK, Cooper TA (2012). Pre-mRNA splicing in disease and therapeutics. Trends Mol. Med..

[30] Pelava A, Schneider C, Watkins NJ (2016). The importance of ribosome production, and the 5S RNP-MDM2 pathway, in health and disease. Biochem. Soc. Trans..

[31] Gruss OJ, Meduri R, Schilling M, Fischer U (2017). UsnRNP biogenesis: mechanisms and regulation. Chromosoma.

[32] Minakhina S, Changela N, Steward R (2014). Zfrp8/PDCD2 is required in ovarian stem cells and interacts with the piRNA pathway machinery. Development.

[33] Minakhina S, Naryshkina T, Changela N, Tan W, Steward R (2016). Zfrp8/PDCD2 interacts with RpS2 connecting ribosome maturation and gene-specific translation. PLoS One.

[34] Poole AR, Vicino I, Adachi H, Yu YT, Hebert MD (2017). Regulatory RNPs: a novel class of ribonucleoproteins that potentially contribute to ribosome heterogeneity. Biol. Open.

[35] Huang Q (2011). Caspase 3-mediated stimulation of tumor cell repopulation during cancer radiotherapy. Nat. Med..

[36] Ryoo HD, Bergmann A (2012). The role of apoptosis-induced proliferation for regeneration and cancer. Cold Spring Harb. Perspect. Biol..

[37] Clavier A, Rincheval-Arnold A, Baillet A, Mignotte B, Guénal I (2016). Two different specific JNK activators are required to trigger apoptosis or compensatory proliferation in response to Rbf1 in Drosophila. Cell Cycle.

[38] Dichtel-Danjoy ML (2013). Drosophila p53 isoforms differentially regulate apoptosis and apoptosis-induced proliferation. Cell Death Differ..

[39] Zitzmann J (2018). Single-cell cloning enables the selection of more productive *Drosophila* melanogaster S2 cells for recombinant protein expression. Biotechnol. Rep. (Amst.).

[40] Uribe E, Venkatesan M, Rose DR, Ewart KV (2013). Expression of recombinant Atlantic salmon serum C-type lectin in *Drosophila* melanogaster Schneider 2 cells. Cytotechnology.

[41] Katayama H, Nagasu T, Oda Y (2001). Improvement of in-gel digestion protocol for peptide mass fingerprinting by matrix-assisted laser desorption/ionization time-of-flight mass spectrometry. Rapid Commun. Mass Spectrom..

