# Supplementary material for: Srlp is crucial for the self-renewal and differentiation of germline stem cells via RpL6 signals in Drosophila testes
Source: Cell Death Dis. 2019 Apr 1;10(4):294. doi: 10.1038/s41419-019-1527-z (PMC6443671; doi:10.1038/s41419-019-1527-z)
Supplement: Supplementary file 1 — Supplementary materials and methods [file 41419_2019_1527_MOESM1_ESM.docx]

Srlp is crucial for the self-renewal and differentiation of germline stem cells via RpL6 signals in *Drosophila* testes

**Supplementary materials and methods**

**CRISPR/Cas9-mediated genome editing**

*Srlp* mutant was generated by an optimized CRISPR/Cas9-mediated genome editing method as described before [S1]. Two sgRNAs targeting *Srlp* exon regions were designed and transcribed to generate fragment deletion. To score for mutations, all G0 adult flies that developed from injected embryos were crossed to ywR13s (ywR13s=yw; sp/Cyo; MKRS/TM2) flies. After laying eggs, G0 adult flies were then screened for the deletions and code shifting by genomic DNA PCR and sequencing. The G1 progeny from G0 positive flies were crossed to ywR13s flies again, and keep the fly stock with heterozygous genotype (yw; sp/Cyo; Srlp mutant/TM2). Primers for DNA PCR were as follows: *Srlp* mutant F, 5-AACGGTGGCAGCTCTGTAAG-3; *Srlp* mutant R, 5-ACCAGTCCAATGTCGTGGGC-3.

**Genomic DNA Extraction**

Fly genomic DNA was purified via phenol-chloroform extraction. Single flies were homogenized in 400 µL of lysis buffer (1X PBS, 0.2% SDS, and 200 µg/mL proteinase K; Roche) and incubated at 50 °C for 1 h, followed by extraction in 400 µL of phenol-chloroform. The mixture was then centrifuged at 21000 g for 20 min at 4 °C, after which the supernatant was transferred to a new tube. An equal volume of isopropanol was added, and the tube was vortexed thoroughly. The mixture was then kept at -20 °C for at least 1 h, followed by centrifugation at 21000 g for 20 min at 4 °C. The supernatant was removed, and the pellet was washed with 500 µl of 75% ethanol, followed by centrifugation at 21000 g for 5 min at 4 °C. Finally, the pellet was dried for 10 min and re-suspended in 30 µl of DNase-free water.

**Western blot**

Western blot analysis was performed as described previously, with minor modifications [S2]. Briefly, cell lysates were separated by electrophoresis, and then transferred to polyvinylidene difluoride (PVDF) membranes (Bio-Rad, Hercules, USA). The membranes were then blocked in 5% non-fat milk and incubated overnight with the indicated primary antibodies (rabbit anti-HA, 3724, Cell Signaling Technology, 1:1000); mouse anti-Tubulin (AT819, Beyotime, 1:1000, China), washed, and incubated at room temperature for 1 h with horseradish peroxidase (HRP)-conjugated secondary antibodies (Thermo Scientific, Waltham, USA). The protein signals were then visualized by SuperSignal West Femto Chemiluminescent Substrate (Thermo Scientific, Waltham, USA).

**Immunoprecipitation (IP)**

pUAS-attB-3xHA-Srlp and pUAS-attB-RpL6-V5 were made by inserting PCR products of *Srlp* CDS and *RpL6-V5* CDS into pUAS-attB-3xHA and pUAS-attB constructs, respectively. Detailed information has been described in the section of plasmid construction. S2 cells transfected with HA-Srlp and RpL6-V5 were subjected to IP assays. IP were performed as described before [S3]

**Supplementary references**

S1. Ren X, Sun J, Housden BE, Hu Y, Roesel C, Lin S *et al*. Optimized gene editing technology for *Drosophila* melanogaster using germ line-specific Cas9. *Proc Natl Acad Sci U S A* 2013; **110**: 19012–19017.

S2. Kang Y, Zheng B, Shen B, Chen Y, Wang L, Wang J *et al*. CRISPR/Cas9-mediated Dax1 knockout in the monkey recapitulates human AHC-HH. *Hum Mol Genet* 2015; **24**: 7255–7264.

S3. Tong C, Jiang J. Using immunoprecipitation to study protein-protein interactions in the Hedgehog-signaling pathway. *Methods Mol Biol* 2007; **397**: 215–229.
