# Supplementary figures and images for: Srlp is crucial for the self-renewal and differentiation of germline stem cells via RpL6 signals in Drosophila testes
Source: Cell Death Dis. 2019 Apr 1;10(4):294. doi: 10.1038/s41419-019-1527-z (PMC6443671; doi:10.1038/s41419-019-1527-z)

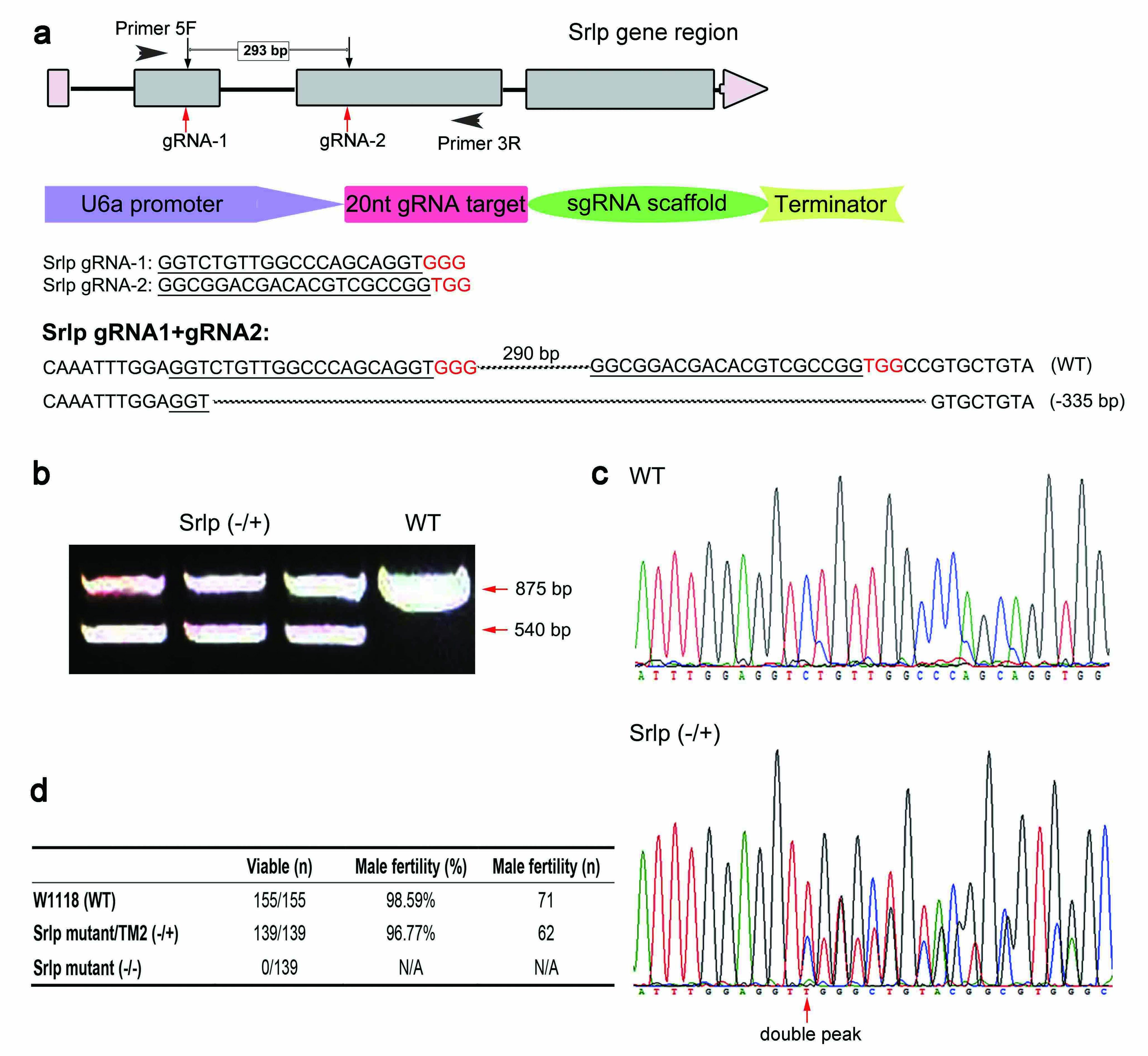

Supplement: Supplementary file 2 — Figure S1 [file 41419_2019_1527_MOESM2_ESM.jpg]

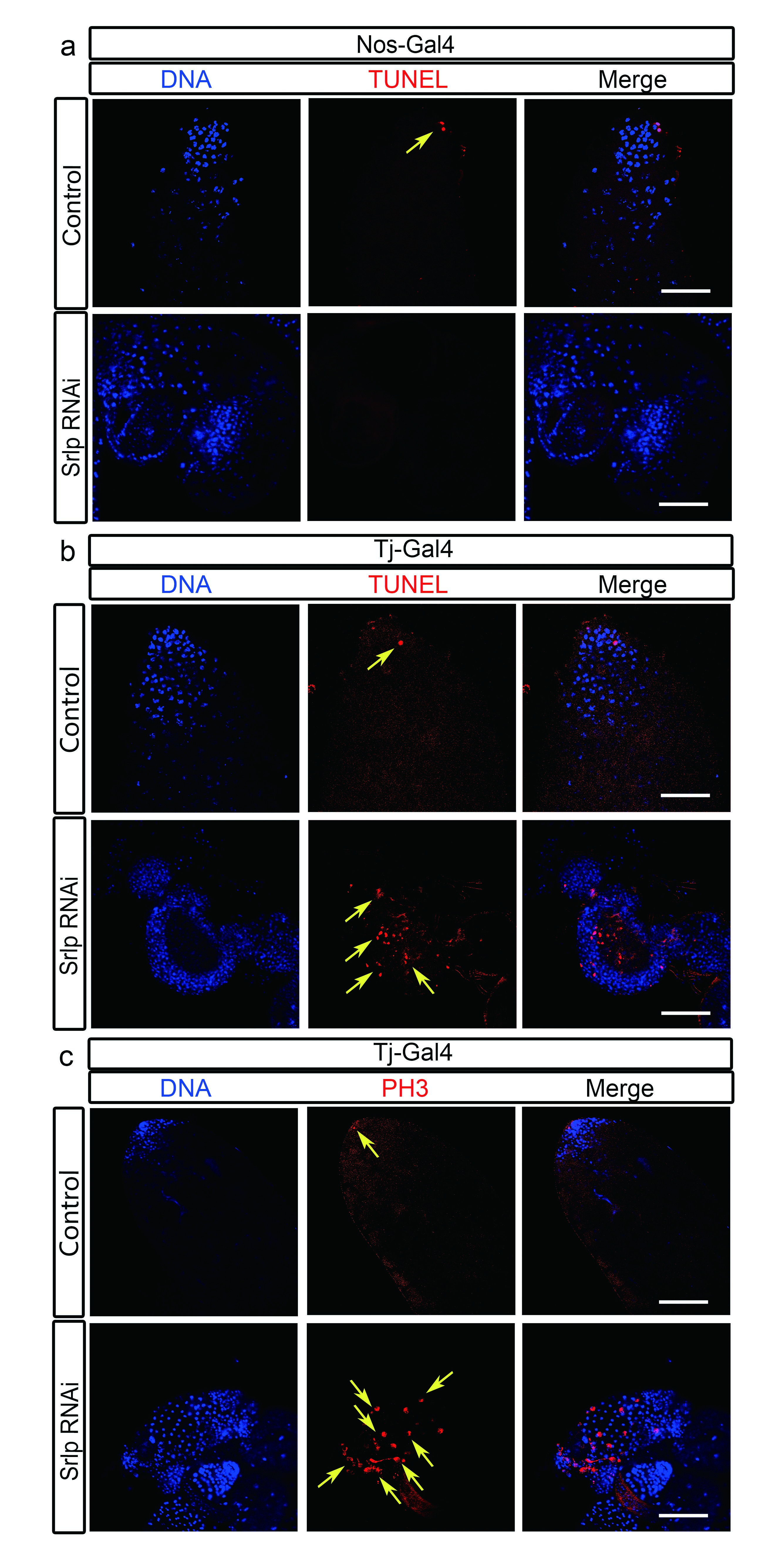

Supplement: Supplementary file 3 — Figure S2 [file 41419_2019_1527_MOESM3_ESM.jpg]

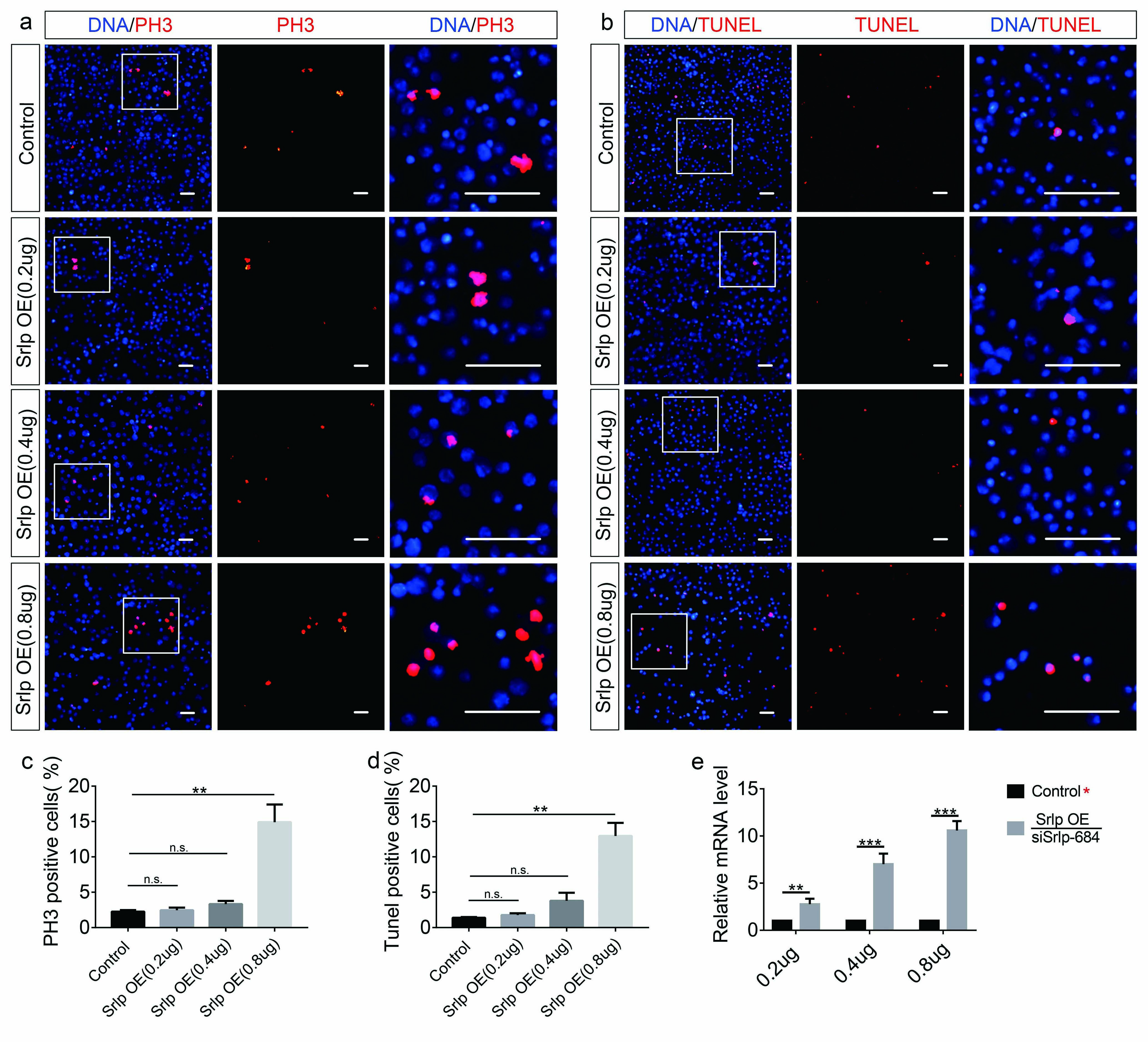

Supplement: Supplementary file 4 — Figure S3 [file 41419_2019_1527_MOESM4_ESM.jpg]

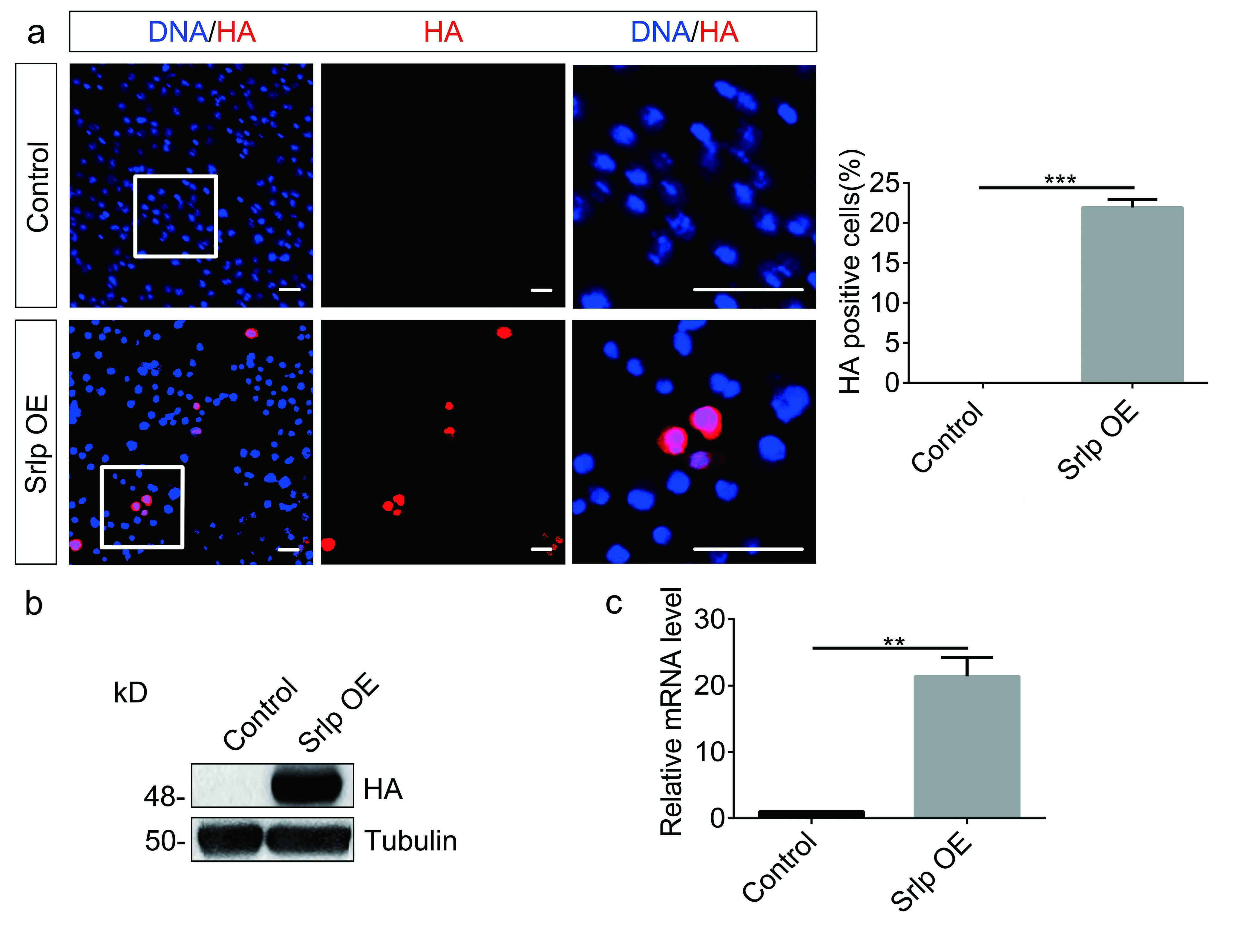

Supplement: Supplementary file 5 — Figure S4 [file 41419_2019_1527_MOESM5_ESM.jpg]

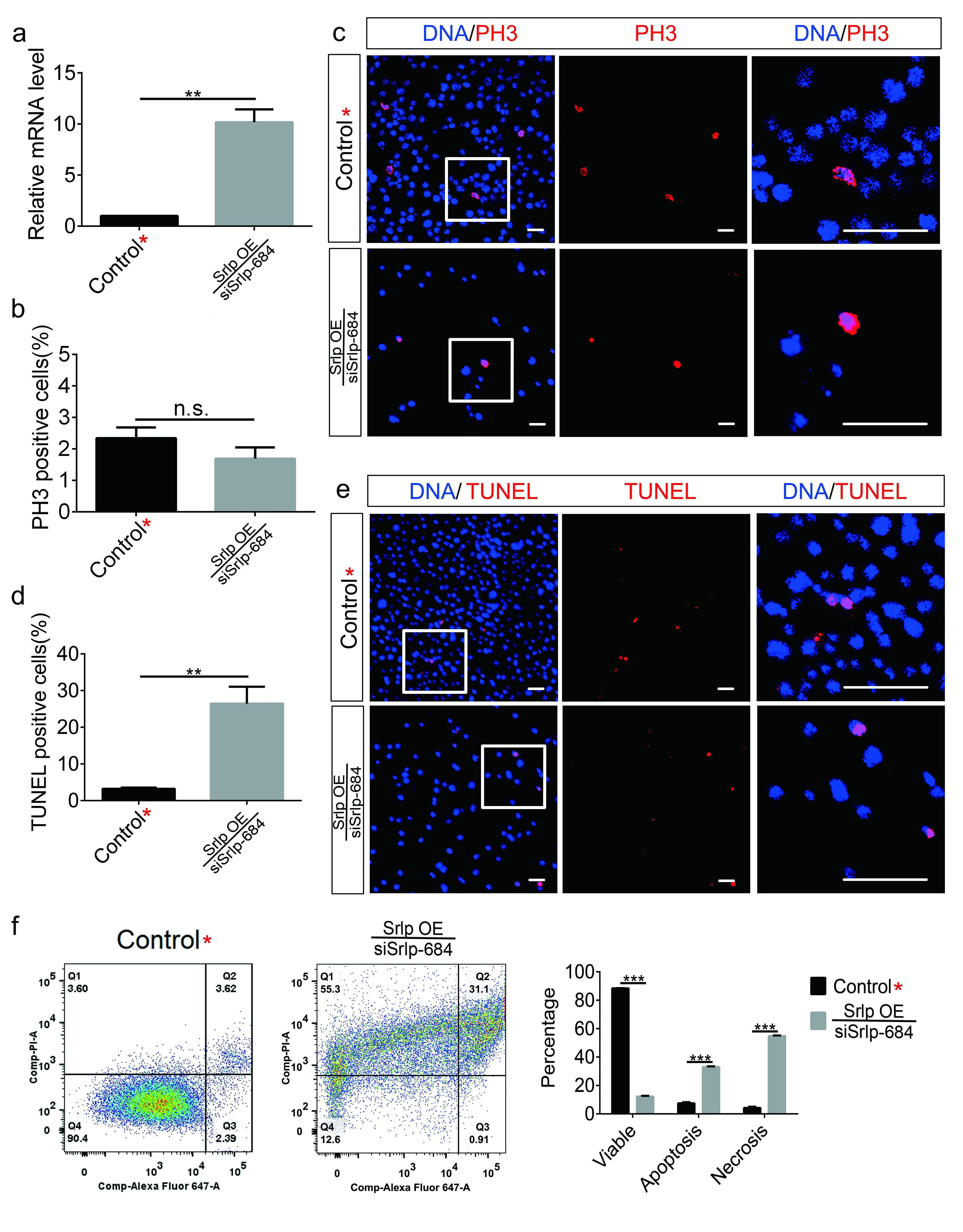

Supplement: Supplementary file 6 — Figure S5 [file 41419_2019_1527_MOESM6_ESM.jpg]

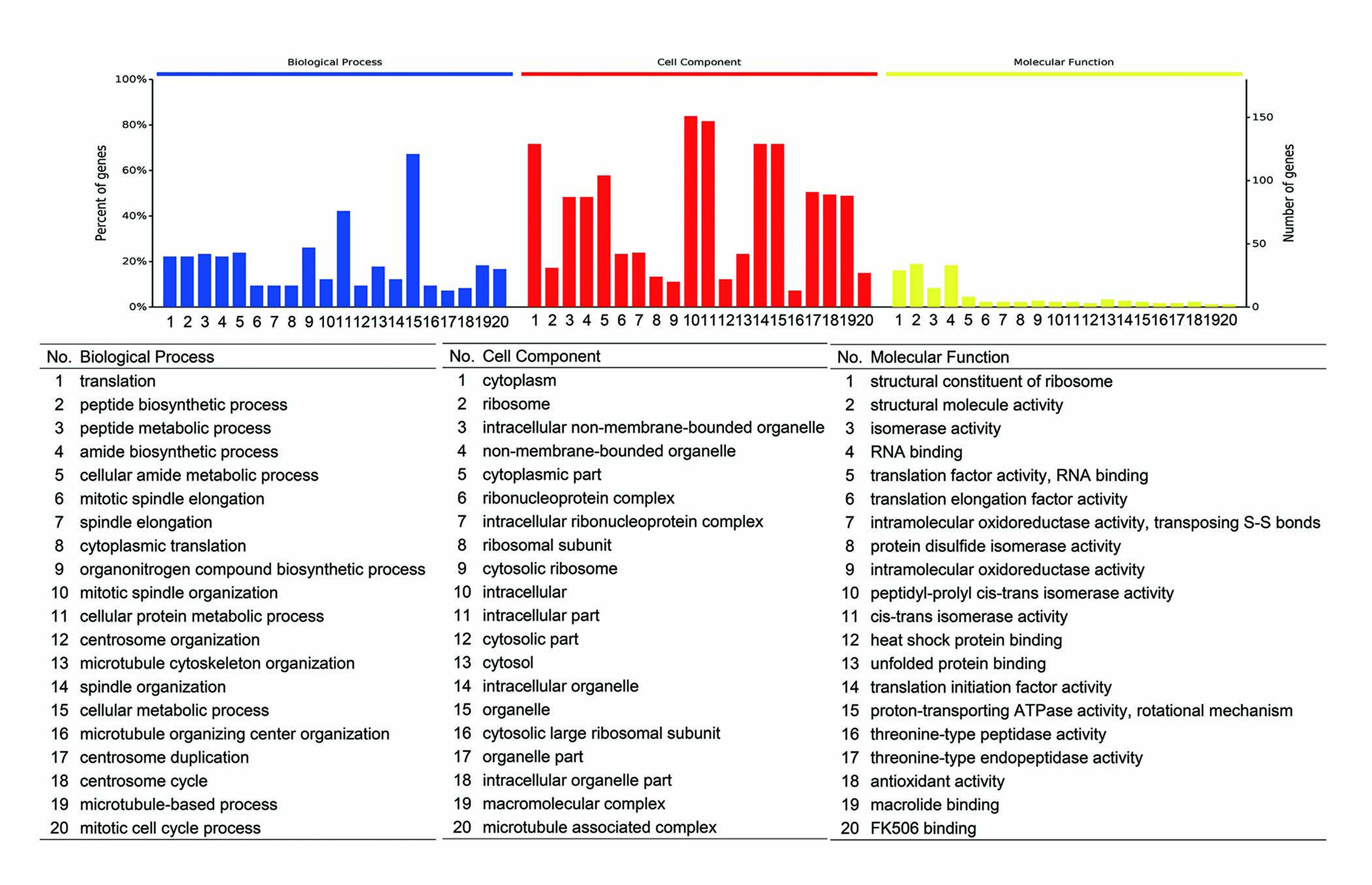

Supplement: Supplementary file 7 — Figure S6 [file 41419_2019_1527_MOESM7_ESM.jpg]

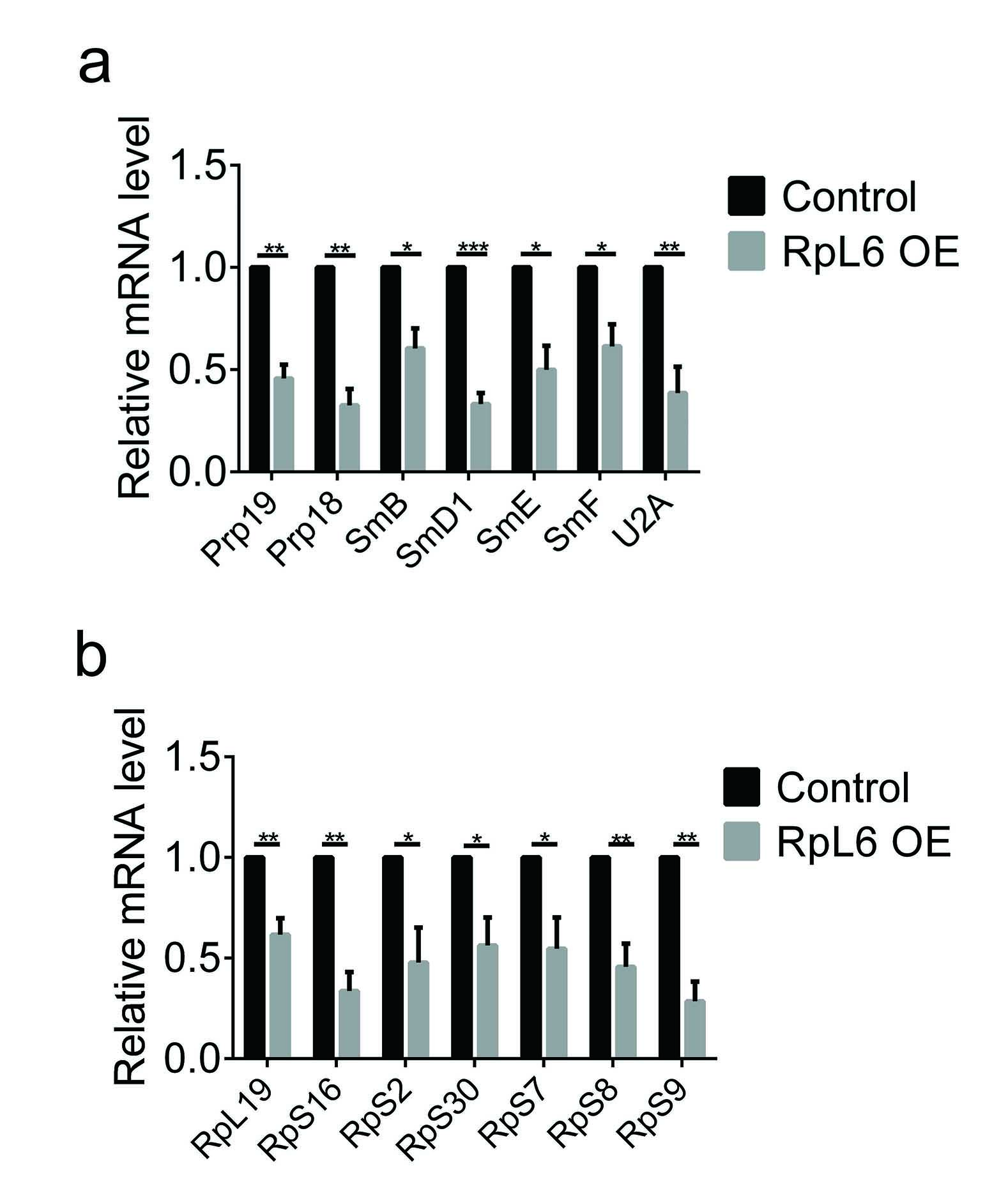

Supplement: Supplementary file 8 — Figure S7 [file 41419_2019_1527_MOESM8_ESM.jpg]

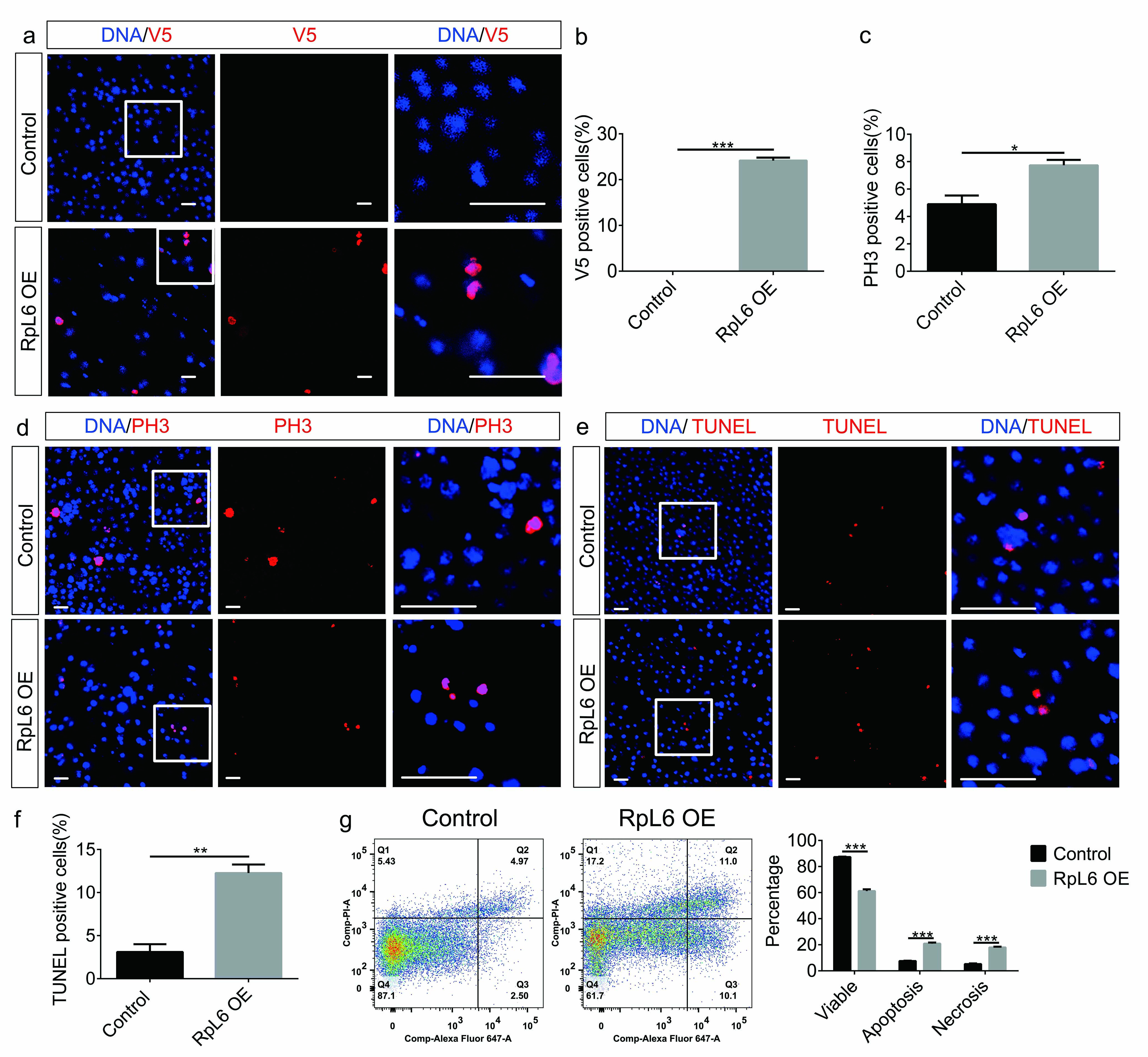

Supplement: Supplementary file 9 — Figure S8 [file 41419_2019_1527_MOESM9_ESM.jpg]

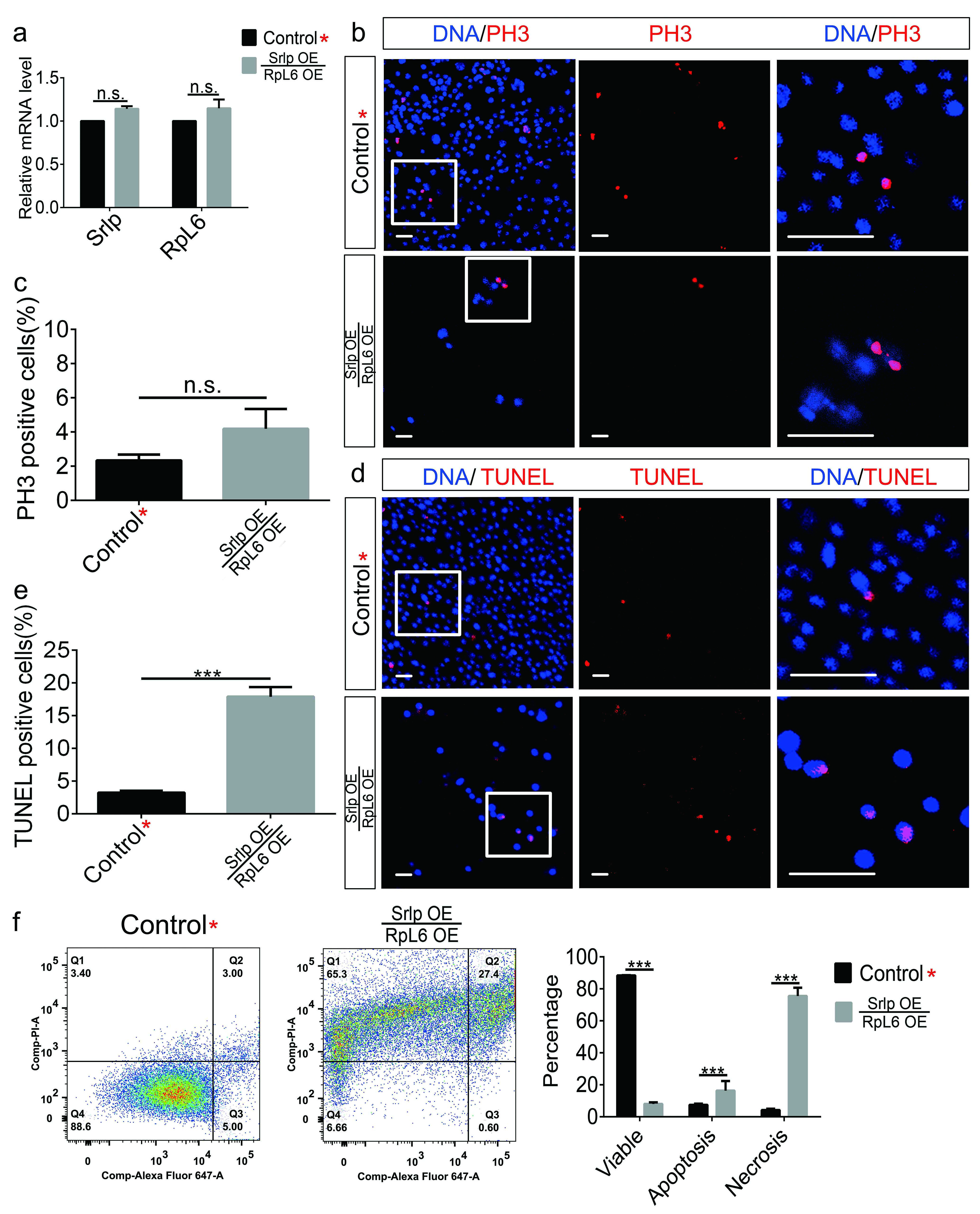

Supplement: Supplementary file 10 — Figure S9 [file 41419_2019_1527_MOESM10_ESM.jpg]

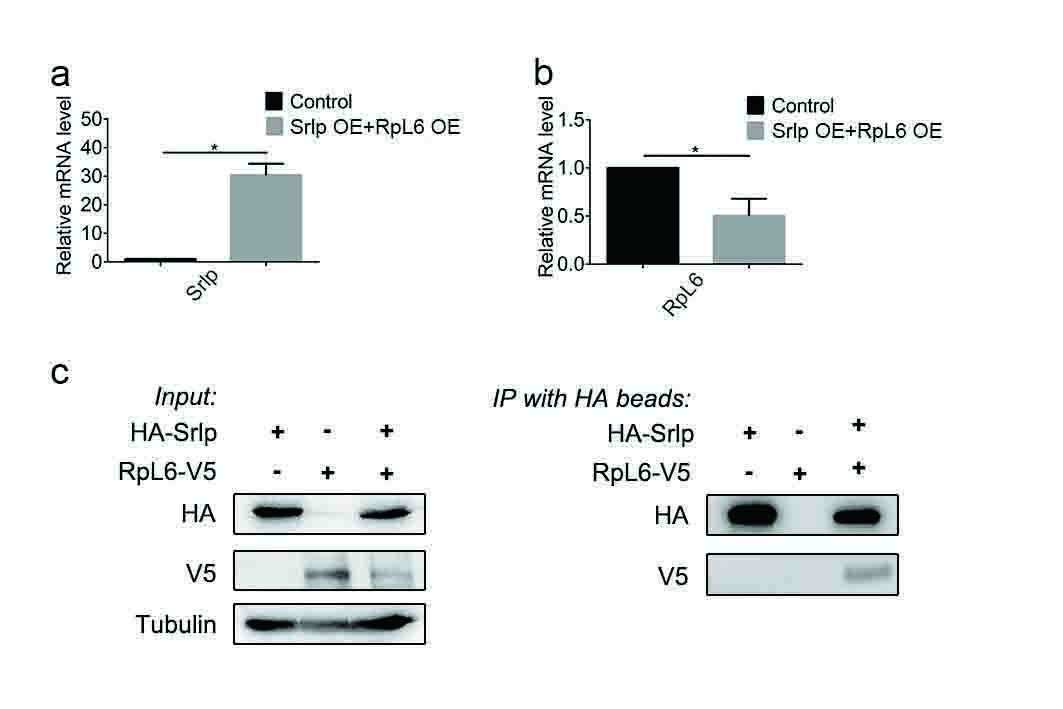

Supplement: Supplementary file 11 — Figure S10 [file 41419_2019_1527_MOESM11_ESM.jpg]
