# Supplementary material for: Srlp is crucial for the self-renewal and differentiation of germline stem cells via RpL6 signals in Drosophila testes
Source: Cell Death Dis. 2019 Apr 1;10(4):294. doi: 10.1038/s41419-019-1527-z (PMC6443671; doi:10.1038/s41419-019-1527-z)
Supplement: Supplementary file 13 — Table S2 [file 41419_2019_1527_MOESM13_ESM.doc]

| **Primer sequences for qRT-PCR.** | | |
| --- | --- | --- |
| **Gene** | **Forward primer(5'-3')** | **Reverse primer(5'-3')** |
| GAPDH | GTGGTGAACGGCCAGAAGAT | GCCTTGTCAATGGTGGTGAA |
| CG5844 | TTAGCACCGACGAGAAGGAGGAG | AGTAGCCATTGATGCCGCACAC |
| Prp19 | GCTGCCACGAAGGACCTGTTAC | CCTGTGCGGATATCGGAGAATGC |
| Prp18 | GCTGCTCACCTTCCTGCTCAAG | AGCGGCTTAACGTATTCCTTAGTCTG |
| SmB | CATGAACTTGATCCTCGGCGACTG | CCTCTGGCGGCGGTGGTC |
| SmD1 | CACCTGAAGAGCGTTCGGATGAC | TGTCGTCGATGAGGAGCGTCTC |
| SmE | CCATCAACCTGATCTTCCGTTACCTG | GCGTCGTCCAGCACCAGATTC |
| SmF | GCTCCGTGACTGGTAATCTTGGC | TCCTCGTCGTCGTCCTCCATG |
| U2A | TCCATCATTCTCACCGGCAACAAC | CTGCTTGATCTTCCTGAAGTCGAGTAG |
| RpL6 | GCACCTGAACGACGCCTACTTC | TCCACCTCCTTCTGGTCCTTCTTG |
| RpL19 | CCTACCAAGCTGCTGTGGATGC | TGCCTGTCAATCTTCTTGCTGTCG |
| RpS16 | AAGGTGAACGGTCGTCCTCTGG | GCTACATGACCACCACCGCTAAC |
| RpS2 | GGCCGGTATTGAGGATTGCTACAC | AGGAAGTCCGAGTATGCCTGGTAAG |
| RpS30 | AGCTGTTCGTCCGTGGACTAGAG | AGTTGGTTCTTGACTCCGGCAATG |
| RpS7 | CTGTGTACGACGCCATCCTTGAG | AATGGTGGTCTGCTGGTTCTTGTC |
| RpS8 | TTGGTGAAGAACAGCATCGTGGTC | CTTGGTCAGCACGTCGTTCTCG |
| RpS9 | TCACATTCGTGTCCGCAAGCAG | TCCTCCTCTTCAGCAGCTCCAC |
