# Supplementary material for: Srlp is crucial for the self-renewal and differentiation of germline stem cells via RpL6 signals in Drosophila testes
Source: Cell Death Dis. 2019 Apr 1;10(4):294. doi: 10.1038/s41419-019-1527-z (PMC6443671; doi:10.1038/s41419-019-1527-z)
Supplement: Supplementary file 14 — Supplementary figure legends [file 41419_2019_1527_MOESM14_ESM.doc]

Supplementary Figure and Table Legends

Figure S1. Generation of *Srlp* homozygous mutant flies. (a) Design of a knockout (KO) strategy for the *Srlp* gene using CRISPR/Cas9 technology. The scheme shows that the design of the gRNA sequence, their location, and indel identified in *Srlp* alleles. (b) PCR detection of *Srlp* deletion and code shifting. (c) Sequencing of the *Srlp* deletion heterozygous mutation. Double peak is shown with red arrow. (d) Viability and fertility rates of the *Srlp* heterozygous and homozygous mutations.

Figure S2. Knockdown of *Srlp* by nos-Gal4 and tj-Gal4 causes tiny testes and dysfunction of germ cells. (a) Immunofluorescence staining and confocal microscopy views of control and nos>Srlp RNAi flies. (b) Immunofluorescence staining and confocal microscopy views of control and tj>Srlp RNAi flies. (c) Immunofluorescence staining and confocal microscopy views of control and tj>Srlp RNAi flies. Immunostaining using TUNEL (red in a and b), PH3 (red in c) and Hoechst 33342 (blue). Scale bar: 50 μm.

Figure S3. Srlp overexpression efficiency in S2 cells. (a) Immunostaining of control and Srlp-overexpressing (0.2μg, 0.4μg and 0.8μg of plasmid in total) S2 cells using anti-PH3 (red) and Hoechst-33342 (blue). (b) Immunostaining of the control and Srlp-overexpressing (0.2μg, 0.4μg and 0.8μg of plasmid in total) S2 cells by TUNEL (red) and Hoechst-33342 (blue). (c) Percentage of PH3-positive cells in the control and Srlp-overexpression group. (d) Percentage of TUNEL-positive cells in the control and Srlp-overexpression groups. (e) Relative *Srlp* mRNA level of control* and Srlp-overexpressing/siSrlp-684 S2 cells. The Student’s *t* test was used for statistical analysis. **P* < 0.05; ***P* < 0.01; ****P* < 0.001. Error bars represent SEM. Scale bar: 30 μm.

Figure S4. HA-Srlp fusion protein is overexpressed in S2 cells. (a) Immunostaining of control and Srlp-overexpressing S2 cells using anti-HA (red) and Hoechst 33342 (blue). And percentage of HA-positive cells in control and Srlp-overexpressing S2 cells was calculated. (b) Western blot analysis of HA in control and Srlp-overexpressing S2 cells. Tubulin was used as internal reference. (c) Relative mRNA level of *Srlp* in control and Srlp-overexpressing S2 cells. Student's *t* test was used. **P*<0.05; ***P*<0.01; ****P*<0.001. Error bars represent SEM. Scale bar: 30 μm.

Figure S5. Rescue experiments of the Srlp overexpression phenotype. (a) Relative mRNA level of *Srlp* of control* and Srlp-overexpressing/siSrlp-684. (b) Percentage of PH3 positive cells in control* and Srlp-overexpressing/siSrlp-684. (c) Immunostaining of control* and Srlp-overexpressing/siSrlp-684 S2 cells by using anti-PH3 (red) and Hoechst 33342 (blue). (d) Percentage of TUNEL positive cells in control* and Srlp-overexpressing/siSrlp-684. (e) Immunostaining of control* and Srlp-overexpressing/siSrlp-684 S2 cells by using TUNEL (red) and Hoechst 33342 (blue). (f) Flow cytometry test of control* and Srlp-overexpressing/siSrlp-684. Ratio of viable cells dramatically decreased, and ratio of apoptosis and necrosis cells significantly increased. Student's *t* test was used. **P*<0.05; ***P*<0.01; ****P*<0.001. Error bars represent SEM. Scale bar: 30 μm.

Figure S6. GO enrichment analysis of identified Srlp binding proteins. Terms related to biological processes, cellular components and molecular functions are shown.

Figure S7. RpL6 overexpression represses the expression level of major spliceosome and ribosome subunits. (a) Relative mRNA level of spliceosome subunits (*Prp18*, *Prp19*, *SmB*, *SmD1*, *SmE*, *SmF*, and *U2A*) in control and RpL6-overexpressing S2 cells. (b) Relative mRNA level of ribosome subunits (*RpL19*, *RpS2*, *RpS7*, *RpS8*, *RpS9*, *RpS16*, and *RpS30*) in control and RpL6-overexpressing S2 cells. Student's *t* test was used. **P*<0.05; ***P*<0.01; ****P*<0.001. Error bars represent SEM.

Figure S8. Effects of RpL6 overexpression in S2 cells. (a) Immunostaining of control and RpL6-overexpressing S2 cells using anti-V5 (red) and Hoechst 33342 (blue). (b) Percentage of RpL6-V5-postive cells in control and RpL6-overexpressing. (c) Percentage of PH3 positive cells in control and RpL6-overexpressing. (d) Immunostaining of control and RpL6-overexpressing S2 cells using anti-PH3 (red) and Hoechst 33342 (blue). (e) Immunostaining of control and RpL6-overexpressing S2 cells using TUNEL (red) and Hoechst 33342 (blue). (f) Percentage of TUNEL positive cells in control and RpL6-overexpressing. (g) Flow cytometry test of control and RpL6-overexpressing. Ratio of viable cells dramatically decreased, and ratio of apoptosis and necrosis cells significantly increased. Student's *t* test was used. **P*<0.05; ***P*<0.01; ****P*<0.001. Error bars represent SEM. Scale bar: 30 μm.

Figure S9. Rescue effects of Srlp overexpression phenotype by overexpressed RpL6 S2 cells. (a) Relative mRNA level of *Srlp* and *RpL6* in control* and Srlp OE/RpL6 OE S2 cells. (b) Immunostaining of control* and Srlp-overexpressing/RpL6-overexpressing S2 cells by using anti-PH3 (red) and Hoechst 33342 (blue). (c) Percentage of PH3 positive cells in control* and Srlp-overexpressing/RpL6-overexpressing. (d) Immunostaining of control* and Srlp-overexpressing/RpL6-overexpressing S2 cells by using TUNEL (red) and Hoechst 33342 (blue). (e) Percentage of TUNEL positive cells in control* and Srlp-overexpressing/RpL6-overexpressing. (f) Flow cytometry test of control* and Srlp-overexpressing/RpL6-overexpressing. Ratio of viable cells dramatically decreased, and ratio of apoptosis and necrosis cells significantly increased. Student's *t* test was used. **P*<0.05; ***P*<0.01; ****P*<0.001. Error bars represent SEM. Scale bar: 30 μm.

Figure S10. RpL6 is a Srlp binding protein in *Drosophila*. (a) Relative mRNA expression level of *Srlp* in control and Srlp-overexpressing + RpL6-overexpressing S2 cells (b) Relative mRNA expression level of *RpL6* in control and Srlp-overexpressing + RpL6-overexpressing S2 cells. (c) Co-IP assay for the interaction between Srlp and RpL6 protein. Student's *t* test was used. **P*<0.05; ***P*<0.01; ****P*<0.001. Error bars represent SEM.

Table S1. Full list of identified Srlp binding proteins in *Drosophila* melanogaster.

Table S2. Detailed information of primers used in this study.
